# Comparative Analysis of Genomic Repeat Content in Gomphocerine Grasshoppers Reveals Expansion of Satellite DNA and Helitrons in Species with Unusually Large Genomes

**DOI:** 10.1093/gbe/evaa119

**Published:** 2020-06-15

**Authors:** Abhijeet Shah, Joseph I Hoffman, Holger Schielzeth

**Affiliations:** e1 Department of Animal Behaviour, Bielefeld University, Bielefeld, Germany; e2 Institute of Ecology and Evolution, Friedrich Schiller University Jena, Jena, Germany; e3 German Centre for Integrative Biodiversity Research (iDiv) Halle-Jena-Leipzig, Germany

**Keywords:** Acrididae, comparative analysis, genome size evolution, Gomphocerinae, mobile DNA, insects, repeatome, repetitive DNA, Orthoptera, satellite DNA

## Abstract

Eukaryotic organisms vary widely in genome size and much of this variation can be explained by differences in the abundance of repetitive elements. However, the phylogenetic distributions and turnover rates of repetitive elements are largely unknown, particularly for species with large genomes. We therefore used de novo repeat identification based on low coverage whole-genome sequencing to characterize the repeatomes of six species of gomphocerine grasshoppers, an insect clade characterized by unusually large and variable genome sizes. Genome sizes of the six species ranged from 8.4 to 14.0 pg DNA per haploid genome and thus include the second largest insect genome documented so far (with the largest being another acridid grasshopper). Estimated repeat content ranged from 79% to 96% and was strongly correlated with genome size. Averaged over species, these grasshopper repeatomes comprised significant amounts of DNA transposons (24%), LINE elements (21%), helitrons (13%), LTR retrotransposons (12%), and satellite DNA (8.5%). The contribution of satellite DNA was particularly variable (ranging from <1% to 33%) as was the contribution of helitrons (ranging from 7% to 20%). The age distribution of divergence within clusters was unimodal with peaks ∼4–6%. The phylogenetic distribution of repetitive elements was suggestive of an expansion of satellite DNA in the lineages leading to the two species with the largest genomes. Although speculative at this stage, we suggest that the expansion of satellite DNA could be secondary and might possibly have been favored by selection as a means of stabilizing greatly expanded genomes.

## Introduction

Large fractions of eukaryotic genomes consist of repetitive elements, which vary considerably in their abundance across species (Charlesworth et al. 1994; Lynch and Conery 2003). The repetitive fraction of the genome, known as the *repeatome*, correlates with genome size both within and among species (Lynch 2007) and therefore likely plays a major role in genome size evolution (Charlesworth et al. 1994; Talla et al. 2017). Some repeats, such as transposable elements, spread as selfish elements that do not benefit the host organism (Doolittle and Sapienza 1980; Orgel and Crick 1980). However, repeats are also known to assume functional roles (Shapiro and von Sternberg 2005), such as centromeric satellite DNA, which is necessary for appropriate chromosome pairing during cell division (Hartl 2000; Plohl et al. 2008). Repeat elements have also been associated with genetic innovation and speciation (Ellegren et al. 2012; Feliciello et al. 2015; Maumus et al. 2015), rendering repeatome analysis relevant to understanding the origin and maintenance of biodiversity in general.

A small number of clades have evolved genome size gigantism, including some gymnosperms, amphibians, crustaceans, lungfish, sharks, velvet worms, flatworms, and grasshoppers (Gregory 2018). Despite these independent origins of extreme genome size expansions, most species have rather compact genomes (Gregory 2018). Overall, genome size does not appear to be related to organismal complexity, a disparity that is known as the *C*-value enigma because genome size is typically quantified by the *C* value (the molecular weight of a haploid genome, Gregory 2005). Instead, certain factors or circumstances may have allowed genome sizes to increase in some groups but not in others, although these conditions are in general poorly understood. A comparative analysis of the repeatomes of species with large genomes may therefore shed light on the *C*-value enigma and contribute toward an improved understanding of genome size expansions.

A desirable approach would be to conduct a comparative analysis of assembled and annotated genomes in which specific repetitive elements can be clearly identified. However, it is precisely the repeat content that has hindered the assembly of reference genomes for species with large genomes (Plohl and Meštrović 2012; Ruiz-Ruano et al. 2016). The largest genomes published so far are draft genomes of the migratory locust *Locusta migratoria* (6.38 Gb, Wang et al. 2014), Norway spruce *Picea abies* (19.6 Gb, Nystedt et al. 2013), and Mexican axolotl *Ambystoma mexicanum* (32.39 Gb, Nowoshilow et al. 2018). The case of the migratory locust illustrates the difficulty of assembling large and repetitive genome sequences, as the current assembly is fragmented into >550,000 scaffolds with an N50 of 322 kb, despite only 12 chromosomes contributing to the species’ large genome size (Wang et al. 2014). The difficulty of assembling repetitive regions in particular has hampered progress in the analysis of repetitive elements in such species.

Recent comparative studies on genome sizes in insect have focused on the entire group at large and included the migratory locust as the only orthopteran with the largest genome in the sample (Petersen et al. 2019; Wu and Lu 2019). Here, we use a comparative approach to study repeat content in a group of grasshoppers that has genome sizes exceeding that of the migratory locust. We chose to study grasshoppers of the subfamily Gomphocerine (Orthoptera, suborder Caelifera, family Acrididae) because they have highly variable genome sizes, both across and in some cases within species (Schielzeth et al. 2014; Gregory 2018; Jetybayev et al. 2018). This clade hosts the largest genomes among all insects and, even across all organisms, it represents one of only a small set of clades with extremely large genomes (Gregory 2018). Although this makes genome assembly challenging for orthopterans, it offers an outstanding opportunity for a comparative analysis of the repeatome.

The short-horned grasshoppers (Caelifera) have a rather conserved basic karyotype with 9 or 12 chromosome pairs (John and Hewitt 1966), so that genome size variation across species are largely due to differences in the sizes rather than the numbers of chromosomes. At the same time, grasshoppers often vary intraspecifically in chromosome number (Palestis et al. 2004). Supernumerary chromosomes (B chromosomes) and chromosomal segments consist mostly of heterochromatin, which is rich in repeats, especially satellite DNA (Ruiz-Ruano et al. 2017; Ruiz-Ruano et al. 2018). Consequently, grasshoppers show stark contrasts between phylogenetically conserved karyotypes, substantial variation in chromosome size, and facultative variation in dispensable DNA segments. The frequent presence of large pieces of additional DNA also suggests that mechanisms of genome size control are rather weak and/or that tolerance to increases in genome size is high.

**Fig. 1.**
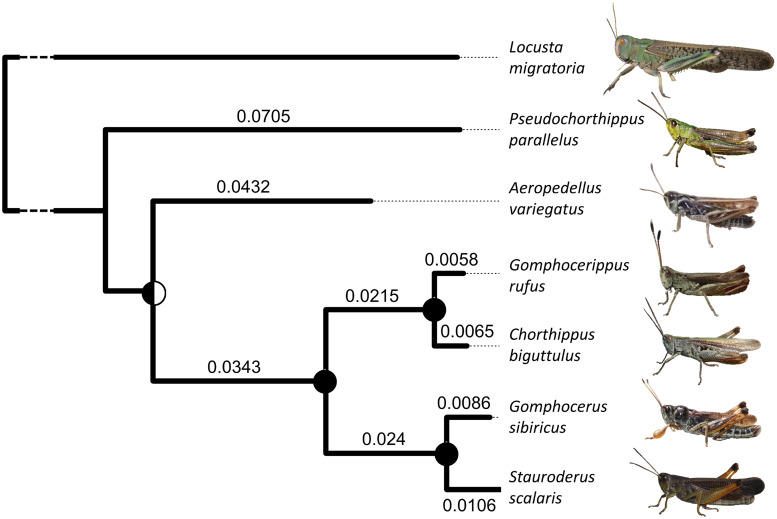
Phylogenetic relationships among six species of gomphocerine grasshoppers (tree rooted using *Pacris xizangensis* as an outgroup) with *Locusta migratoria*, a species used for comparison in some analyses, added with unestimated branch length (the divergence time from gomphocerine grasshoppers is ∼61 Ma, Song et al. 2015). This phylogeny was based on mitochondrial markers (using COI, COII, and COIII genes). Numbers show branch lengths and pie charts at nodes show bootstrap support. The topology is congruent with COI mitochondrial sequence-based analyses published by Vedenina and Mugue (2011) and Dumas et al. (2010).

We used whole-genome shotgun sequencing to characterize the repeatomes of six species of gomphocerine grasshoppers (fig. 1). With low-coverage sequencing it is unlikely that sequences with single copies in the genome will be represented multiple times in the data. Repeated sequences with hundreds or thousands of copies, however, are represented by multiple reads even when sequencing coverage is low. Comparative de novo assembly of low-coverage sequences therefore facilitates the assembly of the repetitive fraction of the genome and thus provides insights into the types and distributions of repetitive DNA. We used a multi-stage analytical pipeline incorporating graph-based de novo clustering of repeat elements (supplementary fig. S1, Supplementary Material online) building on the software packages RepeatExplorer (Novák et al. 2013) and dnaPipeTE (Goubert et al. 2015) as well as RepeatMasker (Smit et al. 2015) and RepBase (Bao et al. 2015) for annotation.

We recently analyzed the repeat content of one species of gomphocerine grasshopper, the club-legged grasshopper *Gomphocerus sibiricus* (Shah et al. 2016). The distribution of repeat types across read clusters of transposable element copies differed markedly from other published distributions (e.g., Piednoel et al. 2012; Lower et al. 2017; da Silva et al. 2018) in that this species shows a large dominance of one particular cluster annotated as satellite DNA. The existence of one predominant class of repeats argues for a recent expansion of this type of repeat sequence in the focal genome, because with an ancient expansion, we would have expected the repeat sequences to have diverged by mutation, which would result in them assembling into multiple clusters rather than into a single cluster. One motivation for the current analysis was therefore to determine whether satellite DNA repeats also appear at high frequency in the genomes of related grasshopper species.

We tested the prediction that grasshopper repeatomes show a strong phylogenetic signal, being more similar in closely related species, while also searching for particular repeat classes showing signs of expansion or reduction in specific lineages. Furthermore, we aimed to evaluate if the unusual pattern of striking dominance of satellite DNA in the genome of *G. sibiricus* is species-specific or represents a more general characteristic of gomphocerine grasshoppers. By analyzing a suite of species that vary substantially in their genome sizes, we aimed to test for a relationship across species between genome size and repeat content. Finally, by analyzing sequence divergence within clusters, we attempted to evaluate the relative ages of expansions of particular repeat classes.

## Materials and Methods

### Species and Sample Collection

We sampled hind legs from one male and one female each of six species from the subfamily Gomphocerinae of acridid grasshoppers (total *n *=* *12 individuals): Meadow grasshoppers *Pseudochorthippus parallelus* (Bielefeld, Germany), alpine thick-necked grasshopper *Aeropedellus variegatus* (Engadin, Switzerland), rufous grasshopper *Gomphocerippus rufus* (Engadin, Switzerland), bow-winged grasshopper *Chorthippus biguttulus* (Bielefeld, Germany), club-legged grasshopper *G. sibiricus* (Engadin, Switzerland), and large mountain grasshopper *Stauroderus scalaris* (Engadin, Switzerland). Based on previous mitochondrial analyses (Dumas et al. 2010; Vedenina and Mugue 2011) as well as our own results (fig. 1), *sibiricus*-*scalaris* and *biguttulus*-*rufus* appear to be sibling taxa, whereas *parallelus* and *variegatus* are more distantly related. Hind legs were stored in 70% ethanol at –20 °C prior to DNA extraction from postfemur muscle tissue using a standard chloroform-isoamyl alcohol extraction protocol (Sambrook et al. 1989).

### Genome Size Determination by Flow Cytometry

We quantified genome sizes by flow cytometry following a standard protocol (Hare and Johnston 2011). Nuclei were extracted from heads of three male grasshoppers per species. Preliminary analyses have shown that freezing after nuclei isolation leads to blurred peaks in the flow cytometer. Therefore, all samples were processed immediately before measurement. Half a brain, split longitudinally, was used per extraction. First, 1 ml of cold Galbraith buffer was added to each sample. Samples were then ground with 15 strokes of a pestle in a Dounce grinder. Both the grinder and pestle were washed with Milli-Q water between the processing of each sample. Homogenates were transferred to Eppendorf tubes and left to incubate for 15 min. Ground samples were filtered through a 20 µm nylon mesh filter to remove cell debris and the filtrate was recovered into a 5 ml falcon tube on ice. 20 µl (5% of the total volume) of the standard *Acheta domesticus* extract was added to each sample. Each extract was further diluted with 100 µl of 0.5 mg/ml propidium iodide to obtain a final concentration of 50 µg/ml. Samples were left to stain for 1 h on ice in the dark before being filtered again using a 20 µm nylon mesh filter and then analyzed on a BD FACS Canto II flow cytometer. Analyses continued at a medium flow rate until 10,000 gated events were recorded.

Flow cytometry data were processed using the BD FACSDiva software. Besides the pronounced peak of the cricket size standard, we usually observed a smaller peak at approximately twice the signal intensity that was putatively caused by mitotically dividing cells. A second peak at twice the signal intensity of the target sample was also sometimes visible, but the peak was small and usually blurred, so that it could not be analyzed. However, these results demonstrate overall linearity of the signal across the observed range. We converted signal intensities to genome sizes by taking the least squares fit of published genomes sizes (averages available for four species, supplementary table S3, Supplementary Material online) on signal intensity (adjusted *R*^2^ = 0.82, supplementary fig. S14, Supplementary Material online).

### High Throughput Sequencing and Short-Read Preprocessing

We generated separate sequencing libraries for all 12 individuals using an Illumina Nextera DNA library preparation kit and size-selected fragments ranging from 300 to 700 bp. These libraries were then 2× 300 bp paired-end sequenced on the Illumina MiSeq sequencing platform, which resulted in 4.5 Gb of sequence and an average depth of coverage across the entire genome of ∼0.0034×. To further increase the quantity of data, we sequenced the same samples with 150 bp single-end reads on two Illumina HiSeq 2500 lanes to yield 31.1 Gb of sequence, corresponding to an average depth of coverage of ∼0.23×. The resulting raw reads were preprocessed and filtered using trimmomatic (version 0.36, Bolger et al. 2014) and FASTX toolkit (version 0.06, Gordon and Hannon 2010) to remove sequencing adapters, sequencing artefacts and low-quality reads (<20 phred). Trimmomatic was set to remove sequencing adapters, leading and low-quality bases (below quality 3), bases which fall below quality 15 in a 4 bp wide window and reads with final lengths below 120 bp.

### Phylogenetic Analysis

We used MitoFinder (version 1.2, Allio et al. 2019), a pipeline to extract and assemble mitochondrial genome from sequencing data, to harvest as many mitochondrial sequences as possible from all samples. Although nuclear sequences would be preferable for phylogenetic reconstruction, our low-coverage sequencing does not yield sufficient coverage of well-represented nuclear genes. Nevertheless, mitochondria are present in higher copy numbers than nuclear mitochondrial copies (which frequently cause problems for phylogenetic analysis in orthopterans, Song et al. 2014; Hawlitschek et al. 2017) and are therefore ideally suited for phylogenetic analysis. We used MAFFT (version 7.313, Katoh and Standley 2013), with the L-INS-i option to create a multiple sequence alignment of mitochondrial genes. We reconstructed phylogenies on a gene-by-gene basis for 15 mitochondrial genes (supplementary fig. S11, Supplementary Material online). Since many genes had missing sequences for some samples, we selected the COI, COII, and COIII genes, which had the least missing data, for a final analysis in which multiple sequence alignments were concatenated (supplementary fig. S10, Supplementary Material online). *Pacris xizangensis* (Li et al. 2020) was added as an outgroup for rooting. The phylogenetic analysis was performed using PartitionFinder (version 2.1.1, Lanfear et al. 2016) in order to select best-fitting partitioning schemes and models of molecular evolution, followed by a maximum-likelihood based phylogeny estimating using RAxML (version 8.2.12, Stamatakis 2014), with a GTR substitution model and GAMMA rate heterogeneity across sites.

### De Novo Repeat Identification

We used RepeatExplorer (version 0.9.7.8) for de novo repeat identification (Novák et al. 2013). Clustering was based on read similarity across multiple copies of repeat elements and in the ideal case, clusters represent all reads from a family of repeats. RepeatExplorer relies on RepeatMasker (version 4.06, Smit et al. 2015), RepBase (version 20160829, Bao et al. 2015), and Dfam (version 2.0, https://dfam.org/help/tools) for identification of repeat families. Initially we did this separately for each sample based on HiSeq reads. As RepeatExplorer can handle only a limited number of reads, we randomly selected 10% of the reads from each sample. This process was repeated five times but the replicate runs yielded virtually identical results, so we present only data from a single RepeatExplorer run per sample (fig. 3).

We conducted an independent analysis to confirm our results from RepeatExplorer using dnaPipeTE (version 1.3, Goubert et al. 2015), an alternative pipeline for the de novo assembly, annotation and quantification of transposable elements. We ran dnaPipeTE with default settings and five Trinity iterations. dnaPipeTE is a fully automated pipeline to assemble and quantify repeats, which assembles repeats from short-read data using the Trinity de novo transcriptome assembler in an iterative fashion. This is followed by annotation of the assembled contigs using RepeatMasker and the RepBase database. Finally, BlastN is used to estimate the relative abundance of transposable elements, to shed light on the transposable element divergence landscape, and to further annotate the assembled unannotated contigs.

### Iterative Repeat Identification and Filtering

We used a custom version of satMiner (Ruiz-Ruano et al. 2016) to filter the sequence data for reads associated with repetitive elements and to estimate the total repeat content per sample. The 12 libraries and the MiSeq and HiSeq reads were processed separately at this stage, resulting in 24 satMiner runs. satMiner uses RepeatExplorer to analyze a small subset of each library (set to 300,000 reads) in order to identify repeat clusters de novo. The fraction of reads assigned to repeat clusters was then used to query the remainder of the sequences. Sequences of high similarity were assigned to newly identified clusters and removed from the pool of sequences before progressing with the next iteration of satMiner by parsing a new subset of 300,000 reads from the remaining pool of reads to RepeatExplorer.

We ran satMiner for five iterations, which involved six de novo assembly steps and five mapping and filtering steps. As satMiner does not retain reads which are assigned to clusters, we modified the code so that this information was retained. Our modified version of satMiner is available via https://github.com/abshah/satminer. To facilitate downstream analyses, the MiSeq read pairs were merged using PEAR (version 0.9.10, Zhang et al. 2014). We then used custom Linux shell scripts to collate MiSeq and HiSeq reads revealing homology to repeat clusters identified by satMiner into a single readsets, which we refer to as “repeat-enriched readsets.”

Again, we used the dnaPipeTE pipeline as an independent method to analyze repeat-enriched readsets. We ran dnaPipeTE with default settings with the number of Trinity iterations set to 5 on all repeat-enriched readsets. Results of repeat-enriched readsets were similar to the dnaPipeTE analysis of full readsets before enrichments (see above) and we therefore present only the former.

### Repeat Content Estimation

The five successive satMiner iterations were used to estimate the total repeat content of each sample. During each iteration *i*, we quantified the percentage of the reads that was de novo assigned to clusters, *p_i_*. We then searched for the set of reads *q_i_* that showed sequence similarity to reads in *p_i_*. As reads that are assigned to clusters (*p_i_*) or that show sequence similarity to reads within clusters (*q_i_*) was sequentially removed, we expected this fraction to decline progressively with each iteration. However, we found that *p_i_* remained approximately constant across iterations, while querying the remaining pool of reads gave rapidly diminishing yields of repetitive sequences *q_i_* (supplementary fig. S3, Supplementary Material online). This suggests that the query step was not fully efficient and that each iteration rediscovered the same repeat clusters rather than finding new ones. In fact, the sum of the fraction filtered out of the total pool and the fraction assigned de novo to clusters quickly stabilized after two iterations (supplementary fig. S3, Supplementary Material online). We therefore used the sum Σ(*p *+* q*) calculated after the last satMiner iteration to provide the best estimate of total repeat content.

### Joint Repeat Clustering and Comparison across Species

Comparing clusters across species can sometimes be difficult due to issues with merging clusters across independent runs in different readsets. Consequently, we analyzed readsets that contained reads from different individuals and species in equal proportions as described below. We processed the repeat-enriched readsets using RepeatExplorer (version 0.9.7.8, Novák et al. 2013). In order to ensure equal representation of repetitive elements from all biological samples, we subsampled each of the twelve enriched readsets 20 times without replacement, each time drawing 25,000 MiSeq reads and 75,000 HiSeq reads at random to produce a total subsample of 100,000 reads per readset. This generated 20 data sets, each comprising 1,200,000 subsampled reads pooled over all 12 individuals that were analyzed by RepeatExplorer to generate de novo assembled repeat clusters.

We then used reciprocal BLAST to match contigs from clusters identified by RepeatExplorer pairwise across independent runs. We aimed to pool the 15 most abundant repeat classes that we assumed to be represented in all runs. As rank order may change across runs, we used the first 50 clusters produced by each run to determine pairwise matches (of which the first 30 are shown in supplementary table S5, Supplementary Material online). Within the pool of 50 × 50 reciprocal BLAST matches across 50 clusters from each of two runs, there was a single best match for the most abundant 15 clusters in all cases (supplementary table S6, Supplementary Material online). Reads from clusters identified as best matches were pooled and the 15 clusters with the most reads across pooled samples were further processed.

We used PCA to compare the overall pattern of repeat clusters across individuals. This was based on the 15 most abundant clusters keeping the 20 replicated sampling draws as independent cases as they contained no overlapping reads. The PCA was therefore performed on 15 items (clusters) and 240 cases (20 replicated subsamples each of 12 individuals). We performed the PCA with variance-standardized items, thus giving all clusters equal weight in the analysis. The first three axis showed eigenvalues above unity and thus explained more variance than any of the original clusters alone. Analyzing only the first ten clusters yielded qualitatively similar results (with two eigenvalues above unity).

Furthermore, we identified reads from different biological samples by visualizing aggregations of reads from different species in different regions of the cluster graphs. Cluster graphs were built on the repeat-enriched pool across all samples and we thus refer to this approach as “pool-and-paint” cluster painting.

### Cluster Annotation

Cluster contigs were annotated by RepeatMasker using the Metazoan database of repeats from RepBase (version 20160829, Bao et al. 2015). dnaPipeTE uses RepeatMasker and RepBase database for annotation and we used BlastN to further annotate the assembled unannotated contigs (supplementary table S7, Supplementary Material online). Annotating de novo assembled clusters is challenging and not all annotations are likely to be correct. Nevertheless, most of our analyses relied on relative cluster sizes and the distribution of reads from clusters across samples, and so were not dependent on accurate annotations.

### Ancestral State Reconstruction

We used ancestral state reconstruction to estimate changes in repeat abundances separately for the major repeat clusters in our set of species. Topology and branch lengths were based on our mitochondrial phylogenetic tree. Repeat abundance was estimated from our RepeatExplorer analysis by multiplying the proportion of reads assigned to each cluster with the estimated genome size of each species. This resulted in an estimate of total sequence content per cluster for each sample. Estimates for males and females were highly correlated and were therefore averaged in the analysis. We then implemented ancestral state reconstruction using REML fits based on a Brownian motion model (as implemented in the ace function of R package ape, version 5.3, Paradis and Schliep 2019) to estimate ancestral states for each node. These were subsequently converted to changes per branch in Mb of sequence per haploid genome.

### Comparative Analysis of the Migratory Locust

For some of our analyses, we also incorporated published sequence data from the migratory locust *L. migratoria*, the only acridid species (from the subfamily Oedipodinae) for which a draft genome has been published (Wang et al. 2014). Raw paired-end Illumina HiSeq 2000 sequences (73.6 Gb) were downloaded from the short-read archive (accession number SRR764584 and SRR764591). We merged read pairs using PEAR (version v0.9.10, Zhang et al. 2014) to create a readset with long single-end reads for comparability with our analysis of gomphocerine species described above. Merged reads below 60 bp were removed. We did not combine reads from *L. migratoria* with reads from the six gomphocerine species in our pooled RepeatExplorer analysis because the species is too distantly related and would distort the pattern of interspecific variation.

## Results

We combined low coverage short-read sequencing with graph-based clustering to characterize the relative abundances of the most common repeats across six species of gomphocerine grasshoppers (fig. 1). For brevity, and because genus assignment has recently been in flux, we hereafter refer to each taxon only by its species name (*parallelus*, *variegatus*, *biguttulus*, *rufus*, *sibiricus*, and *scalaris*, respectively). Genome size was determined by flow cytometry using the house cricket *A. domesticus* as a size standard (2.1 pg DNA per haploid genome). We found that genome size varied across species by a factor of 1.7, with *scalaris* having the largest genome (∼14.0 pg) and *biguttulus* the smallest (∼8.4 pg, supplementary fig. S2, Supplementary Material online). Sequencing of 12 individuals, comprising one individual of each sex from six different species, resulted in a total of ∼311 million reads, which after quality filtering was reduced to ∼300 million reads (20.4–43.0 million reads per sample) totaling 34.1 Gb of data (supplementary table S1, Supplementary Material online).

### Repetitive Content and Genome Size

We estimated the size of the repetitive fraction of each individual’s genome based on five satMiner iterations as described in the Materials and Methods section. The fraction *p_i_* of newly discovered repeats declined as iterations *i* progressed but stabilized at a positive value (supplementary fig. S3, Supplementary Material online). In total, satMiner identified between 2,376 and 5,544 contigs per sample. The fraction of reads *q*_i_ that matched repeat clusters increased per iteration and then stabilized (supplementary fig. S3, Supplementary Material online). The sum of these two fractions represents an estimate of the total repeat content. This was highly correlated between the two sexes of the same species (*r *=* *0.96, *t*_4_* *=* *6.56, *P *=* *0.0028) and variable among species, with *biguttulus* showing the lowest repeat content (79%) and *scalaris* the highest (96%, supplementary fig. S3, Supplementary Material online). Applying the same procedure to reads from the published *Locusta* genome (Wang et al. 2014) resulted in an estimated repeat content of 71%. Alternative quantifications by a single RepeatExplorer run and based on dnaPipeTE yielded lower, but highly correlated estimates for our set of six species (supplementary table S2, Supplementary Material online).

**Fig. 2.**
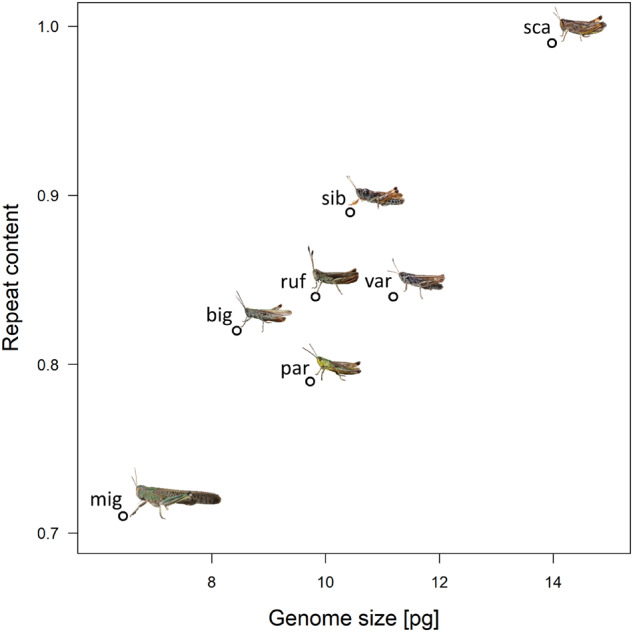
Relationship between repeat content as estimated by de novo clustering (see supplementary fig. S3, Supplementary Material online) and genome size as estimated by flow cytometry (see supplementary fig. S2, Supplementary Material online) for six species of gomphocerine grasshoppers and *Locusta migratoria*. par = *Pseudochorthippus parallelus*, var = *Aeropedellus variegatus*, ruf = *Gomphocerippus rufus*, big = *Chorthippus biguttulus*, sib = *Gomphocerus sibiricus*, sca = *Stauroderus scalaris*, mig = *L. migratoria*.

Genome size quantification was performed using flow cytometry and compared with the three species for which published genome sizes are available (supplementary table S3, Supplementary Material online). Our estimates were similar to previous publications for *scalaris* (13.98 vs. 14.72), lower for *parallelus* (9.73 vs. 12.31) and higher for *sibiricus* (10.43 vs. 8.95). Both these cases might represent population differences, because our measurements were taken from other populations than previous estimates (supplementary table S3, Supplementary Material online). Total repeat content was strongly and positively correlated with genome size across species (gomphocerine species only: *r *=* *0.87, *t*_4_ = 3.62, *P *=* *0.022, including *Locusta*: *r *=* *0.93, *t*_5_ = 5.70, *P *=* *0.0023, Pearson’s correlation test, fig. 2).

### Characterization of Repeat Content within Species

Averaged across species dnaPipeTE annotated ∼24% of the repeatome as DNA transposons, 13% as helitrons, 21% as LINE elements, 12% as LTR retrotransposons, 1.6% as SINE elements, 8.5% as satellite DNA, and 19% as low-copy number elements (supplementary figs. S4 and S5, Supplementary Material online). There was marked variation of the relative proportions of these different repetitive elements among species. Particularly pronounced was the large abundance of satellites in *sibiricus* and *scalaris* and the low abundance of satellites in *parallelus* (supplementary figs. S4 and S5, Supplementary Material online). Helitrons were found to be quite common in all species, but were most abundant in *scalaris* (supplementary figs. S4 and S5, Supplementary Material online). Other repeat classes were less variable among species in their relative abundances.

**Fig. 3.**
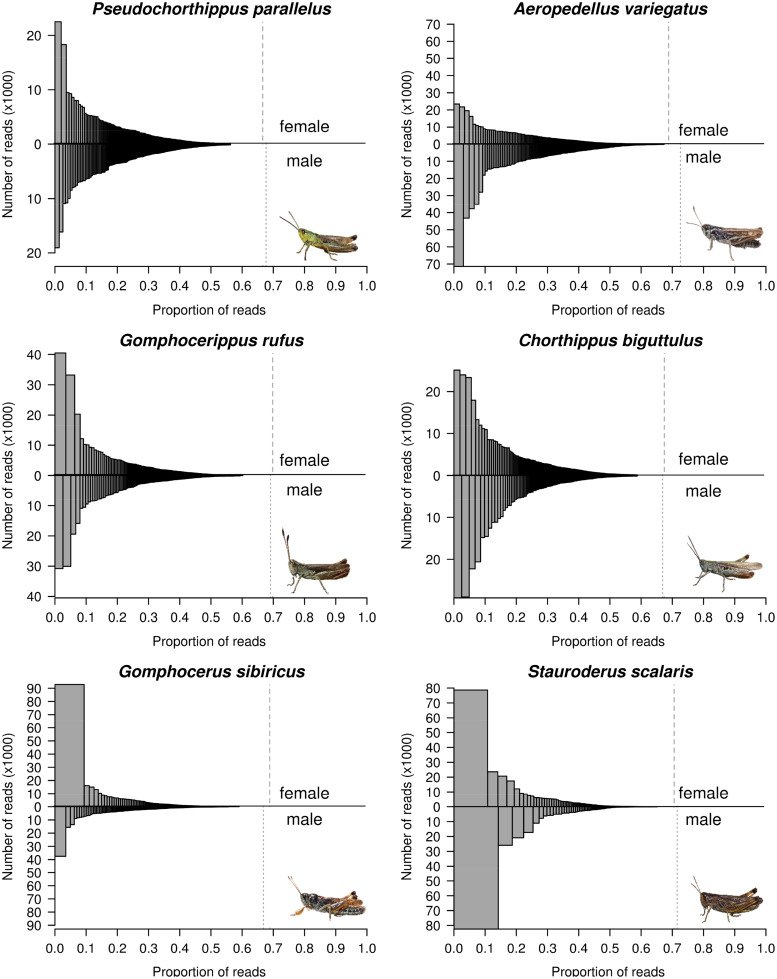
Distribution of de novo assembled repeat content over repeat clusters. The upper half of the plot shows results for the female sample whereas the lower half shows the male sample. Each histogram is based on a single clustering run, with other runs being qualitatively similar. Dashed vertical lines show the estimated repeat content for males and females as estimated by RepeatExplorer based on this single run.

When assembling the repeatome de novo using RepeatExplorer, we found a “tapering” pattern of repeat cluster frequencies in all species and in both sexes (fig. 3). In most species, there was no markedly dominating cluster of repeats. A similar pattern was present in *Locusta* (supplementary fig. S6, Supplementary Material online). However, a strikingly different pattern was obtained for *scalaris* as well as for the female *sibiricus* individual, both of which appear to be dominated by a single highly abundant cluster. In these species, the most abundant cluster accounted for ∼10–15% of the total number of reads. In all samples of *scalaris*, *sibiricus*, and *biguttulus*, as well as in the *variegatus* male, the most abundant cluster was annotated as satellite DNA, whereas in all other cases the top cluster was either annotated as helitrons or could not be annotated.

### Divergences within Clusters of Transposable Elements

We estimated the average divergences within read clusters of transposable element copies using dnaPipeTE (supplementary figs. S7 and S8, Supplementary Material online). Sequence divergence was highest for SINE elements (6.9%) and DNA transposons (6.3%), intermediate for helitrons (5.9%) and LINE elements (5.4%), and lowest for LTR retrotransposons (4.2%). Variation in sequence divergence across species was low for DNA transposons, LINE elements, and LTR retrotransposons, but pronounced for helitrons (lowest in *scalaris*, 4.8%; >5.7% in all other species) and SINE elements (lowest in *scalaris*, 4.5%; >6.5% in all other species).

### Variation in Repeat Content across Species

While the sample-by-sample analysis provided an unbiased picture of repeat content distribution within samples, matching clusters across samples was less straightforward. We therefore conducted an additional analysis in which we pooled reads across samples and collectively de novo assembled their repeat content. We extracted the first 15 repeat clusters (constituting 12–37% of the genome per sample) and analyzed how reads of different samples contributed to these clusters. We found strong positive correlations in repeat content between the two samples from the same species (average Person correlation *r *=* *0.94 across the first 15 clusters, supplementary fig. S9, Supplementary Material online) implying that the two biological replicates within each species were highly similar and that intraspecific differences were low compared with interspecific variation.

**Fig. 4.**
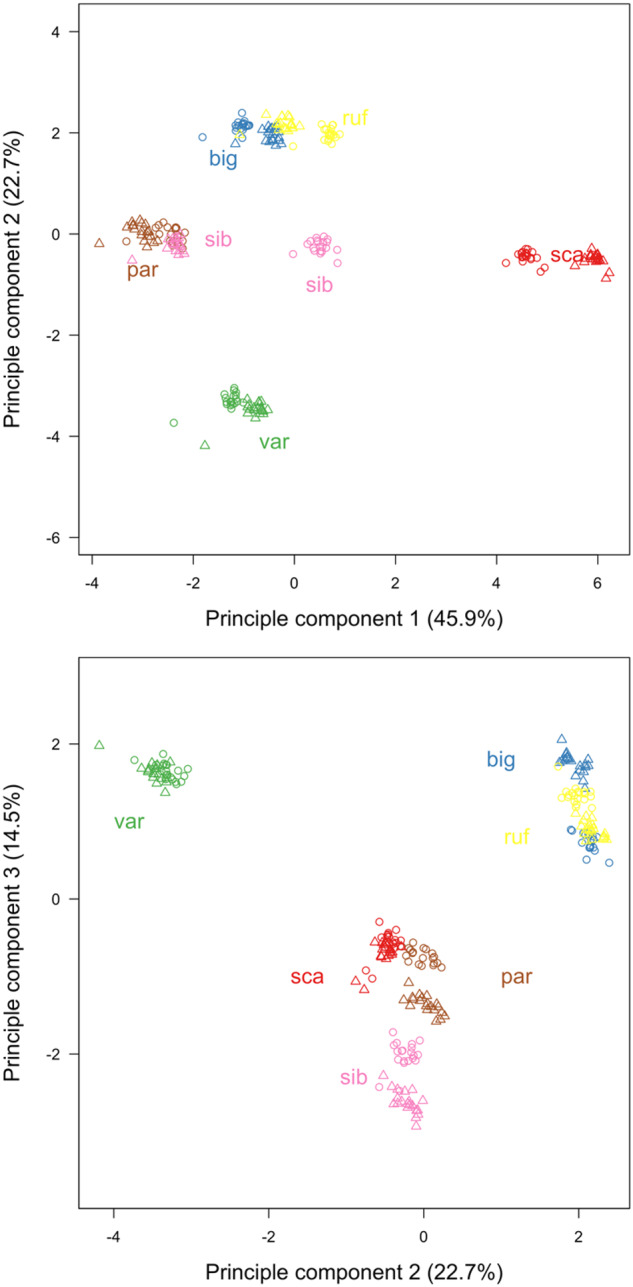
Principle component analysis of repeat content (based on the 15 most abundant clusters) across six species of gomphocerine grasshoppers using variable scaling and rotation of axes. The first three principle components explain 48%, 25%, and 15% of the variation, respectively. Each point represents the results of a single run, with species distinguished by color, females shown as circles and males as triangles. par = *Pseudochorthippus parallelus*, var = *Aeropedellus variegatus*, ruf = *Gomphocerippus rufus*, big = *Chorthippus biguttulus*, sib = *Gomphocerus sibiricus*, sca = *Stauroderus scalaris*.

**Fig. 5.**
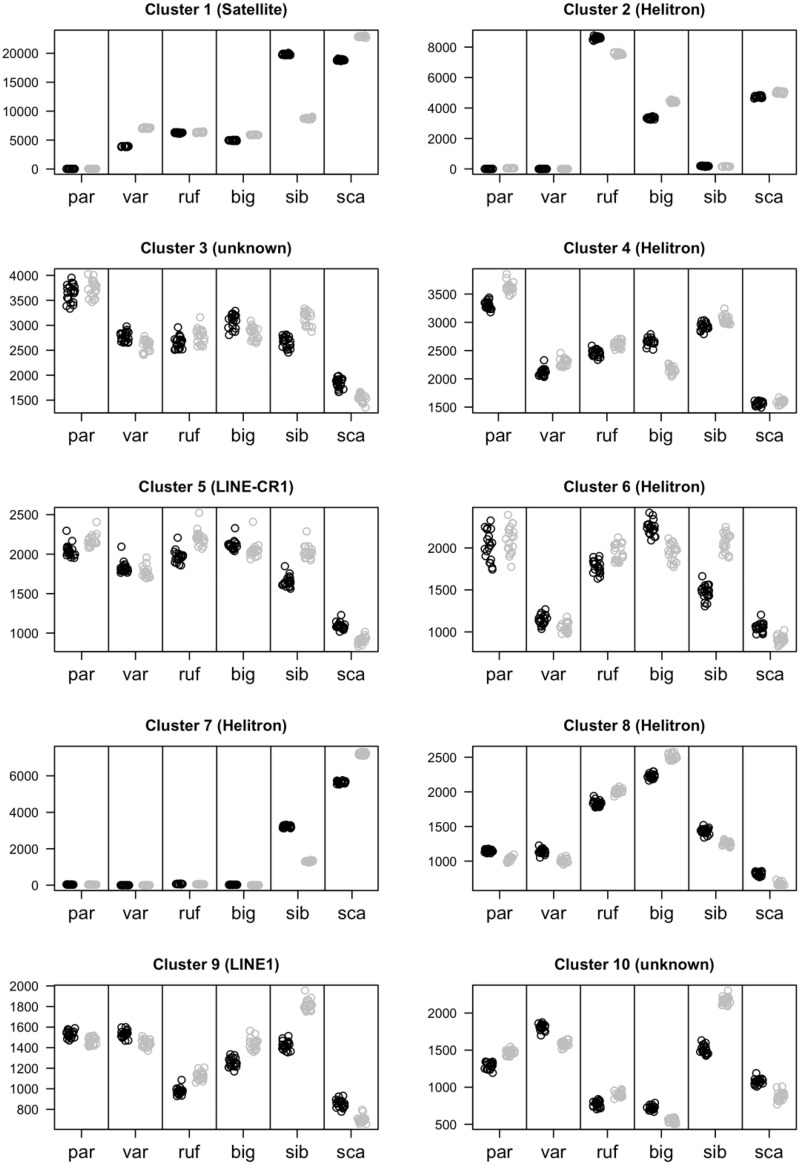
Abundance of the ten most abundant repeat clusters across six species of gomphocerine grasshoppers. Species are arranged horizontally according to their phylogenetic relatedness, as shown in figure 1. Females are shown in black and males are shown in gray. Each dot represents one of twenty independent clustering runs based on nonoverlapping subsets of the data. par = *Pseudochorthippus parallelus*, var = *Aeropedellus variegatus*, ruf = *Gomphocerippus rufus*, big = *Chorthippus biguttulus*, sib = *Gomphocerus sibiricus*, sca = *Stauroderus scalaris*.

To visualize the distribution of repeat clusters both within and among species, we conducted a principle component analysis (PCA) focusing on the 15 most abundant clusters that could be matched across runs. Three main patterns emerged (fig. 4). First, all runs from the same sample clustered tightly together, illustrating that our subsample size was sufficiently large to robustly estimate among-sample variation. Second, samples of females and males from the same species also clustered closely together, except for the two *sibiricus* individuals, which showed a marked intraspecific difference in PC1 values. Third, related species tended to cluster together, in particular the species pair *biguttulus*/*rufus*. To investigate these patterns further, we plotted the frequencies of the most abundant clusters separately for males and females of all species (fig. 5). Variation within *sibiricus* was found to arise mainly from differences in the abundance of the satellite cluster (cluster 1) although the female also had a higher frequency of cluster 7 (helitrons) and the male had a higher frequency of clusters 6, 9, and 10 (helitrons, LINE1 elements, and unnamed, respectively).

### Intraspecific Differences in *G. sibiricus*

The male *sibiricus* sample was unusual in several aspects (cluster size distribution, fig. 3; PCA, fig. 5; sequence divergence within clusters, supplementary table S4, Supplementary Material online). However, three lines of evidence suggest that these patterns were not simply caused by sample mix-up, sequencing artefacts or contamination, since 1) both independent MiSeq and HiSeq runs yielded similar patterns, 2) the samples of the two *sibiricus* individuals clustered together in our phylogenetic reconstruction based on mitochondrial reads (supplementary figs. S10 and S11, Supplementary Material online), and 3) BLAST queries against standard databases did not yield any unusual hits. Nevertheless, we placed more confidence in the female *sibiricus* sample because of the better match with independent samples analyzed previously (Shah et al. 2016).

For among species comparisons, the characteristic feature of the *rufus*/*biguttulus* pair was the high abundance of helitrons of clusters 2 and 8 and the low abundance of cluster 10. *Scalaris* showed a particularly high abundance of satellites (cluster 1) and helitrons of cluster 7. *Parallelus* and *variegatus* as the two most divergent species in our data set showed rather different distributions, with *variegatus* being an outlier in the PCA (fig. 4) and *parallelus* in the abundance of clusters 1–4 (fig. 5). *Parallelus* was characterized by a low abundance of satellites (cluster 1) and helitrons of clusters 2 and 7, but a relatively high abundance of helitrons from clusters 4 and 6. *Variegatus* was different in being rather average in representation across clusters. Mapping changes in clusters size across the phylogeny using ancestral state reconstruction provided tentative evidence for increases in satellites (cluster 1), helitrons (cluster 7), simple repeats (cluster 15), and unknown (cluster 3) from the most ancestral species (*parallelus*/*variegatus*) to the most derived species (*sibiricus*/*scalaris*), but also some apparent decreases in cluster sizes, such as for helitrons of cluster 11 (supplementary fig. S12, Supplementary Material online). Strongest positive correlations between repeat abundance and genome size were found for cluster 1 (satellite), cluster 7 (helitron), and cluster 15 (simple repeats) (supplementary table S8, Supplementary Material online).

### Species Differences Explored by Cluster Painting

**Fig. 6.**
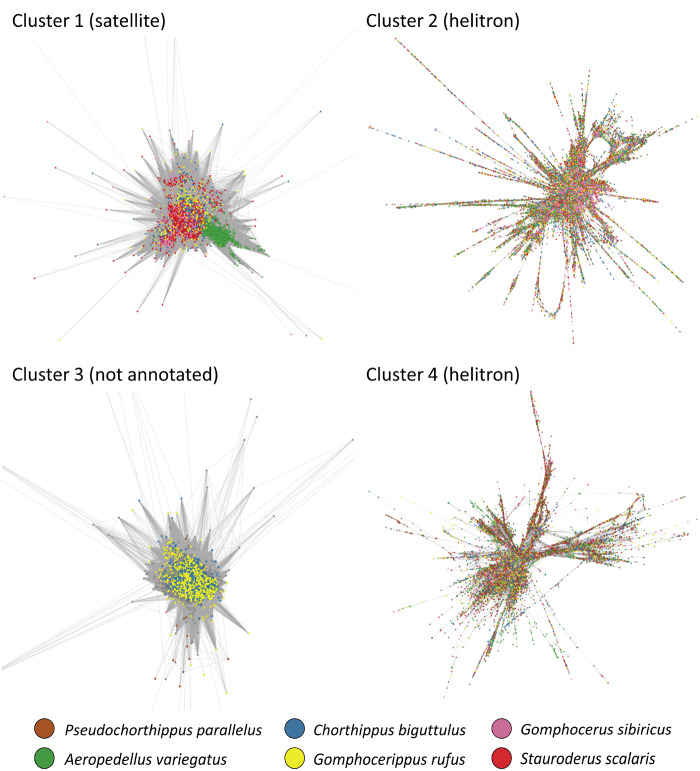
Cluster-paining approach to species-specific differences within cluster. The plot shows the four largest clusters with dots representing reads and read overlap by edges. The six different species are shown by different colors. Tight clustering of reads from the same species (as for clusters 1 and 3) indicate divergence within a species, whereas dispersion of colors across the graph (as for clusters 2 and 4) indicates that either cluster expansion predates divergence or expansion has continued from a range of diversified repeat copies.

Reads within clusters (as identified by RepeatExplorer) can be visualized as graphs in which individual reads are represented by nodes and read overlaps by edges. If a given repeat class spread prior to the split of two species, we would expect reads of those species to be distributed randomly across graphs due to sequence divergence prior to and after the species split. By contrast, if a repeat class expanded and diverged after the split of two species, we would expect reads from the same species to cluster together within graphs. We therefore color-coded reads by sample in the joint graph in an approach that can be described as “pool-and-paint” cluster painting (fig. 6, supplementary fig. S13, Supplementary Material online). We found that clusters 1 (annotated as satellite DNA) showed closer relationships of reads within species as opposed to between species (fig. 6), indicating sequence divergence after species split. Clusters 3 and 7 showed similar tight clustering of reads from *biguttulus* and *rufus* that both covered similar regions of the graph (fig. 6, supplementary fig. S13, Supplementary Material online). In contrast, clusters 2, 4–6, and 9–10 showed a much more even distribution of samples across graphs (fig. 6, supplementary fig. S13, Supplementary Material online), suggesting that the divergence is older such that diversity is shared among species.

## Discussion

We here present a comparative analysis of the repeat content of six species of gomphocerine grasshoppers, including *S. scalaris*, which has the second largest insect genome described to date (Gregory 2018). We found a large fraction of retrotransposons, in particular LINEs and LTRs but few SINEs, and a relative high abundance of satellite DNA and helitrons. We also found substantial variation in repeat content among species, whereas marked intraspecific differences were only found in *G. sibiricus*. The distribution across repeat classes was evenly skewed in most of the species, apart from *sibiricus*/*scalaris*, where a single repeat class was dominant, indicative of a recent expansion of satellite DNA in these two species or their common ancestor. The remaining species exhibited a relatively even distribution of repeat classes, suggesting that invasion by repeats is either ancient or that multiple repeat types spread simultaneously in the more recent past. The latter conclusion is supported by the relatively young and unimodal distribution of divergence times within clusters.

Repeat content varied between 79% and 87% across most of the species, the only exception being *scalaris*, which had an estimated repeat content of 96%. Overall, there was a strong positive correlation between repeat content and genome size as described elsewhere (Charlesworth et al. 1994; Talla et al. 2017; Petersen et al. 2019; Wu and Lu 2019). The repeat content in *Locusta* (genome size 6.44 pg) was estimated at 71% using our method, which linearly prolongs the positive correlation between genome size and repeat content. Repetitive elements are thus likely drivers for genome size expansion, possibly due to positive feedbacks that allow these elements to spread more easily in large genomes (Hollister and Gaut 2009). Our asymptotic estimate of repeat content in *Locusta* was slightly higher than that of Wang et al. (2014), possibly reflecting the difficulty of assembling and estimating repeat content through genome assembly (Wang et al. 2014).

One of our most striking results was the expansion of satellite DNA in *sibiricus*/*scalaris*. We suggest that causality might be reversed in this case, in the sense that satellite DNA may not be the cause of genome size expansion, but rather a consequence. Previous studies suggest that satellite DNA may contribute substantially to genome size in grasshoppers with large genomes (Ruiz-Ruano et al. 2016; Shah et al. 2016). Satellite DNA is known to be particularly abundant in the centromeric and telomeric parts of the genome and leads to densely packed heterochromatin structures (Plohl et al. 2008). Centromeric heterochromatin has a function in the pairing of sister chromatids and is therefore important for proper cell division (Hartl 2000; Plohl et al. 2008). It is conceivable that a stabilizing function of satellite DNA might be required when chromosomes become greatly expanded as in the case of grasshoppers. Satellite DNA often evolves in a concerted fashion (Palomeque and Lorite 2008; Plohl and Meštrović 2012; Garrido-Ramos 2017), as indicated in our data by the clustering of reads within species, but different variants of satellite motifs seem to be recruited from a conserved pool of ancestral satellites. Satellite DNA occurs both unclustered and spatially clustered in the genome and it has been suggested that local clusters may have evolved secondarily (Ruiz-Ruano et al. 2016; Palacios-Gimenez et al. 2017). If satellite DNA contributes to chromosome integrity, such expansions might be adaptive in species with large genomes.

Our results also suggest that helitrons have accumulated in gomphocerine grasshoppers. Helitrons spread via rolling circle replication (Thomas and Pritham 2015). They can occur in large numbers (such as in some plants, Xiong et al. 2014) but tend to be rarer than retrotransposons in most animals (Kapitonov and Jurka 2007). Although we also detected many retrotransposons, the relatively high abundance of helitrons in grasshoppers is noteworthy. As with satellite DNA, it is possible that the abundance of helitrons is not the primary cause of genome size expansion, but that they have proliferated in already large genomes. However, relatively high sequence divergence suggests a relatively old age for the spread of helitrons. *Scalaris* represents an exception to the otherwise largely similar representation across species in that helitrons are particularly common in this large-genome species. There are multiple avenues for such positive feedbacks, including more target insertion sites and weaker negative selection per insertion (Hollister and Gaut 2009). Helitrons are biologically significant because they often include fractions of nonhelitron DNA, sometimes entire genes, and thus offer a vehicle for the genomic translocation of functional elements (Thomas and Pritham 2015). Furthermore, helitrons and a number of other transposable elements have been shown to be involved in horizontal gene transfers across insects (Peccoud et al. 2017; Wu and Lu 2019).

In order to visualize interspecific patterns, we mapped species-specific reads to clusters. We used an approach that we describe as pool-and-paint cluster painting to visualize if reads from different samples occupy different parts of the graphs of pooled reads. As we describe above, we pooled reads in order to avoid biases that could arise if we had clustered different libraries independently. Our approach allows shared clusters to appear in the joint analysis even if cluster sizes are small in individual samples. Cluster painting allows explorative assessment, based on the idea that within clusters, reads originating from a recent expansion within a species should cluster more closely together. While this represents an explorative analysis that does not in itself yield a quantitative measure of variation within and among samples, it has the potential to serve as a visualization technique and explorative tool for other applications, particularly when comparing different populations or species. The method relies on sequence differences among lineages and is thus likely to work best for data from rather divergent forms.

Our cluster painting approach showed that reads within cluster graphs were structured by phylogenetic relatedness in at least some cases (fig. 6, supplementary fig. S13, Supplementary Material online). This suggests that repetitive elements often proliferated after lineage splits. However, not all clusters showed such a pattern (e.g., clusters 2, 4–6, and 9–10), suggesting that some elements may have expanded during the earlier phylogenetic history of the Gomphocerinae. The relatively similar sequence divergence within clusters (supplementary figs. S7 and S8, Supplementary Material online) is also suggestive of older expansions, except for LTR retrotransposons, which appear to be younger (supplementary table S4, Supplementary Material online).

*Gomphocerus sibiricus* was the only species for which the distribution of repeats differed markedly between the two samples. In principle, this difference may be driven by the sex chromosomes. *Sibiricus* has three large and five medium-sized pairs of autosomes and the X chromosome is of similar size to the smaller autosomes (Gosálvez and López-Fernández 1981). It also has an X0 sex determination system, in which females have two and males have one copy of the X chromosome. However, as the repeat content of the two sexes did not differ substantially from one another in any of the other species, we consider a sex chromosome explanation unlikely. Alternatively, interindividual differences within species may result from the presence or absence of supernumerary chromosomes (B chromosomes) or supernumerary segments of normal chromosomes, which are facultatively present in some individuals (Gosálvez and López-Fernández 1981). However, the male *sibiricus* sample was unusual in several aspects and also differed markedly from data generated for different individuals of the same species in a recent study (Shah et al. 2016). Consequently, it is possible that this particular sample may be untypical, possibly due to genuine differences in genome structure, or alternatively as a result of unknown biases that could have arisen during the sequencing or assembly procedure. However, the congruence of the two independent library preparations and sequencing runs as well as the results of our mitochondrial phylogenetic reconstruction suggest that these differences probably have a biological rather than technical origin.

Overall, our analysis of repeat content in the large genomes of gomphocerine grasshoppers reveals a strong link between genome size and repeat content, and in particular high abundances of various helitrons and satellite DNA. We suggest that the expansion of satellite DNA might be secondary and could potentially have been favored by selection as a means of stabilizing these greatly expanded genomes. Whether or not helitrons played a primary or secondary role in grasshopper genome size expansions remains an open question, but it seems reasonable to speculate that increases in genome size likely followed a multi-step process, in which different repetitive elements proliferated during the earlier and later phases of genome size expansion.

## Supplementary Material

evaa119_Supplementary_Data

## Notes

**Data deposition:** The short-read data generated in this study have been submitted to the NCBI BioProject database (https://www.ncbi.nlm.nih.gov/bioproject) under the accession number PRJNA559340. Additional files have been deposited at the iDiv data repository (http://idata.idiv.de/ddm/Data/ShowData/1838).
