# Supplementary material for: Comparative Analysis of Genomic Repeat Content in Gomphocerine Grasshoppers Reveals Expansion of Satellite DNA and Helitrons in Species with Unusually Large Genomes
Source: Genome Biol Evol. 2020 Jun 15;12(7):1180–93. doi: 10.1093/gbe/evaa119 (PMC7486953; doi:10.1093/gbe/evaa119)
Supplement: evaa119_Supplementary_Data [file evaa119_supplementary_data.pdf]

Supplementary tables and figures

Comparative analysis of genomic repeat content in grasshoppers reveals expansion of satellite DNA and helitrons in species with largest genomes

Abhijeet Shah, Joseph I. Hoffman, Holger Schielzeth

	Page
**Table S1:** Summary of sequence output.	2
**Table S2:** Comparison of repeat content estimation.	3
**Table S3:** Published genome size estimates.	4
**Table S4:** Sequence divergence within clusters summarized by repeat class and sample.	5
**Table S5:** Matching of clusters by reciprocal blast.	6
**Table S6:** Rank order of matching clusters across runs.	7
**Table S7:** Annotation of repeat clusters.	8
**Table S8:** Pearson correlation of genome size with repeat abundance.	9
**Figure S1:** Flow chart of the data processing procedure.	10
**Figure S2:** Genome size determination by flow cytometry.	11
**Figure S3:** Total repeat content estimation.	12
**Figure S4:** Repeatome classification in females.	13
**Figure S5:** Repeatome classification in males.	14
**Figure S6:** Repeat cluster distribution for *Locusta migratoria*.	15
**Figure S7:** Within-cluster sequence divergence in females.	16
**Figure S8:** Within-cluster sequence divergence in males.	17
**Figure S9:** Consistency of cluster size across sexes.	18
**Figure S10:** Mitochondrial phylogenetic tree by sample.	19
**Figure S11:** Sample-specific gene trees by mitorchondrial gene locus.	20
**Figure S12:** Summary of repeat expansion and compression across phylogeny.	21
**Figure S13:** Cluster-painting display for clusters 5-10.	22
**Figure S14:** Calibration of flow cytometry signal intensity to genome size.	23
**Supplementary references**	24

Table S1: Summary of sequencing output (after quality filtering) in seven species of Acridid grasshoppers. The *Locusta* dataset is a subset of the *Locusta* genome project sequencing runs (Wang *et al.* 2014).

Species	Sex	# PE reads	# SE reads	Total bp	Coverage
Pseudochorthippus parallelus	Male	431,243	26,123,484	3,004,651,879	0.32x
Pseudochorthippus parallelus	Female	368,771	22,047,786	2,538,590,762	0.27x
Aeropedellus variegatus	Male	898,886	42,144,856	4,935,907,687	0.45x
Aeropedellus variegatus	Female	486,548	23,135,739	2,702,135,625	0.25x
Gomphocerippus rufus	Male	510,974	21,326,566	2,520,426,131	0.26x
Gomphocerippus rufus	Female	615,659	22,032,317	2,638,581,869	0.27x
Chorthippus biguttulus	Male	375,586	23,198,795	2,640,534,948	0.32x
Chorthippus biguttulus	Female	374,817	22,850,071	2,612,756,133	0.32x
Gomphocerus sibiricus	Male	463,973	19,980,621	2,376,345,690	0.23x
Gomphocerus sibiricus	Female	511,351	21,892,521	2,589,274,427	0.25x
Stauroderus scalaris	Male	375,939	28,034,183	2,448,458,508	0.18x
Stauroderus scalaris	Female	290,636	21,662,158	3,167,983,886	0.23x
Locusta migratoria	Female	8,420,257	0	7,482,931,189	1.28X

Table S2: Comparison of repeat content estimation by satMiner, RepeatExplorer and dnaPipeTE (correlations estimates satMiner-RepeatExplorer *r* = 0.56, satMiner-dnaPipeTE *r* = 0.93, RepeatExplorer-dnaPipeTE *r* = 0.72).

Species	Sex	satMiner	RepeatExplorer	dnaPipeTE
Pseudochorthippus parallelus	Male	0.79	0.68	0.61
Pseudochorthippus parallelus	Female	0.83	0.69	0.63
Aeropedellus variegatus	Male	0.84	0.73	0.64
Aeropedellus variegatus	Female	0.86	0.70	0.65
Gomphocerippus rufus	Male	0.84	0.69	0.64
Gomphocerippus rufus	Female	0.83	0.67	0.63
Chorthippus biguttulus	Male	0.82	0.67	0.62
Chorthippus biguttulus	Female	0.86	0.69	0.65
Gomphocerus sibiricus	Male	0.89	0.67	0.63
Gomphocerus sibiricus	Female	0.94	0.71	0.68
Stauroderus scalaris	Male	0.99	0.72	0.70
Stauroderus scalaris	Female	0.79	0.68	0.61

Table S3: Published genome size estimates as compiled in the Animal Genome Size Database (http://www.genomesize.com/). DNA content refers to haploid genome size. NA = Information not available.

Species	DNA content [pg]	Method	Cell type	Size standard	Origin	Refs
Pseudochorthippus parallelus	12.31	Fuelgen densitometry	Testes	*Locusta migratoria* (5.5 pg DNA)	UK?	John & Hewitt (1966)
Pseudochorthippus parallelus	13.36	Fuelgen densitometry	Testes	*Mus musculus* (3.3. pg DNA)	UK?	Wilmore & Brown (1975)
Pseudochorthippus parallelus	13.83	NA	NA	NA	NA	Petitpierre (1996)
Pseudochorthippus parallelus	14.72	Fuelgen densitometry	Testes	*Gallus domesticus* (1.25 pg DNA)	Spain	Belda *et al.* (1991)
Chorthippus brunneus	8.55	Fuelgen densitometry	Testes	*Locusta migratoria* (5.5 pg DNA)	UK?	John & Hewitt (1966)
Chorthippus brunneus	8.55	Fuelgen densitometry	Testes	*Locusta migratoria* (5.5 pg DNA)	UK?	Wilmore & Brown (1975)
Chorthippus brunneus	10.15	Fuelgen densitometry	Testes	*Allium cepa* (16.5 pg DNA)	Spain	Gosalvez *et al.* (1980)
Gomphocerus sibiricus	8.95	Fuelgen densitometry	Testes	*Allium cepa* (16.5 pg DNA)	Spain?	Gosalvez *et al.* (1980)
Stauroderus scalaris[1]	14.72	Fuelgen densitometry	Testes	*Gallus domesticus* (1.25 pg DNA)	Spain	Belda *et al.* (1991)
Stauroderus scalaris	16.34	NA	NA	NA	NA	Petitpierre (1996)

[1]Listed as *Chorthippus scalaris*.

Table S4: Sequence divergence within clusters summarized by repeat class and sample as estimated by dnaPipeTE.

Species	Sex	DNA transposons	Helitrons	LINE elements	LTR retro-transposons	SINE elements
Pseudochorthippus parallelus	Female	6.184	6.288	5.204	4.364	8.006
Pseudochorthippus parallelus	Male	6.354	6.463	5.255	4.313	7.995
Aeropedellus variegatus	Female	6.298	6.297	5.260	4.009	6.475
Aeropedellus variegatus	Male	6.293	5.215	5.270	4.071	6.635
Chorthippus biguttulus	Female	6.555	6.313	5.510	4.343	7.840
Chorthippus biguttulus	Male	6.681	6.313	5.522	4.298	7.654
Gomphocerippus rufus	Female	6.390	6.057	5.349	4.205	7.393
Gomphocerippus rufus	Male	6.222	6.167	5.372	4.123	7.518
Gomphocerus sibiricus	Female	6.207	5.872	5.219	4.101	7.193
Gomphocerus sibiricus	Male	7.616	7.996	7.043	7.865	9.245
Stauroderus scalaris	Female	6.168	4.944	5.510	4.012	4.735
Stauroderus scalaris	Male	6.171	4.738	5.387	4.050	4.291

Table S5: Matching of repeat clusters across independent runs here shown by example of runs 1 and 2. Rows show clusters as assigned in run 1 and columns show clusters as assigned in run 2. Numbers in cells show the number of reciprocal blast hits for contigs from each cluster in one run against all contigs from each clusters in the other run. In this particular example, the best-matching clusters for the first 20 clusters of each run are (Run 1-Run 2): 1-1, 2-3, 3-2, 4-4, 5-5, 6-6, 7-7, 8-8, 9-10, 10-13, 11-14, 12-12, 13-18, 14-15, 15-9, 16-17, 17-20, 18-16, 19-20, 20-11, 21-19. Among those, we consider the 17-20 match somewhat ambiguous.

Cluster in run 1 \ Cluster in Run 2	1	2	3	4	5	6	7	8	9	10	11	12	13	14	15	16	17	18	19	20	21	22	23	24	25	26	27	28	29	30
**1**	9	·	·	·	·	·	·	·	·	·	·	·	·	·	·	·	·	·	·	·	·	·	·	·	·	·	·	·	·	·
**2**	·	·	13	·	·	·	·	·	·	·	·	·	·	·	·	·	·	·	·	·	·	·	·	·	·	·	·	1	·	·
**3**	·	480	·	3	·	·	·	·	·	·	·	·	·	1	·	1	·	·	·	·	·	·	·	·	·	·	2	·	·	·
**4**	·	5	·	229	·	1	·	·	·	·	·	1	·	·	1	·	·	1	·	·	·	·	·	·	·	·	·	·	·	·
**5**	·	·	·	·	50	·	·	·	·	·	·	·	·	·	·	1	·	·	·	·	·	·	·	·	·	·	·	·	·	·
**6**	·	1	·	·	·	109	·	·	·	·	·	·	·	1	·	·	·	·	·	·	·	·	·	·	·	·	·	·	·	·
**7**	·	·	·	·	·	·	11	·	·	·	·	·	·	·	·	·	·	·	·	·	·	·	·	·	·	·	·	·	·	·
**8**	·	·	·	1	·	·	·	22	·	·	·	·	·	·	·	·	·	·	·	·	·	·	·	·	·	·	·	·	·	·
**9**	·	·	·	·	·	·	·	·	·	29	·	·	·	·	·	·	·	·	·	·	·	·	·	·	·	·	·	·	·	·
**10**	·	·	·	·	·	·	·	·	·	·	·	·	66	·	·	·	·	1	·	·	5	·	·	·	·	·	·	·	·	·
**11**	·	·	·	·	·	·	·	·	·	·	3	·	·	35	·	·	·	·	·	·	·	·	·	·	·	·	·	·	·	·
**12**	·	·	·	·	·	·	·	·	·	·	·	42	·	·	·	·	·	·	·	·	2	·	·	·	2	·	·	·	·	·
**13**	·	·	·	·	·	·	·	·	·	·	·	·	·	·	·	·	·	26	2	1	·	·	·	·	·	·	·	·	·	·
**14**	·	·	·	·	·	·	·	·	·	·	3	·	·	·	37	·	·	·	·	13	·	·	·	·	·	·	·	·	2	·
**15**	·	·	·	·	·	·	·	·	221	·	·	·	·	·	·	·	·	·	·	·	·	·	·	·	·	·	·	·	·	·
**16**	·	·	·	·	·	·	·	·	·	·	·	·	·	·	·	·	203	·	·	·	·	·	·	·	·	·	·	·	·	·
**17**	·	·	·	·	·	·	·	·	·	·	·	·	5	·	·	·	·	1	·	8	·	·	·	·	·	·	·	·	·	·
**18**	·	1	·	5	·	·	·	·	·	·	·	·	·	·	·	119	·	·	·	·	·	·	·	·	1	·	·	·	·	4
**19**	·	·	·	·	·	·	·	·	·	·	1	·	·	·	3	·	·	·	·	52	·	·	·	·	·	·	·	·	7	·
**20**	·	·	·	·	·	·	·	·	·	·	154	·	·	·	·	·	·	·	·	1	2	·	·	·	·	·	·	·	·	·
**21**	·	·	·	·	·	·	·	·	·	·	·	·	·	·	·	·	·	·	17	·	·	·	·	·	·	·	·	·	·	·
**22**	·	·	·	·	·	·	·	·	·	·	·	·	·	·	·	·	·	·	·	·	·	9	·	·	·	·	·	·	·	·
**23**	·	·	·	·	·	·	·	·	·	·	·	·	·	·	·	·	·	·	·	·	·	·	·	9	·	·	·	·	·	·
**24**	·	·	·	·	·	·	·	·	·	·	·	·	·	·	·	·	·	·	·	·	·	·	6	·	·	·	·	·	·	·
**25**	·	·	·	·	·	·	·	·	·	·	·	·	·	·	·	·	·	·	·	·	·	·	·	·	18	·	·	·	·	·
**26**	·	·	·	·	·	·	·	·	·	·	12	·	·	·	·	·	·	·	·	·	141	·	·	·	·	·	·	·	·	·
**27**	·	·	·	·	·	·	·	·	·	·	·	·	·	·	·	·	·	·	·	·	·	·	·	·	·	30	·	·	·	·
**28**	·	30	·	1	·	·	·	1	·	·	1	1	·	1	·	2	·	·	·	·	·	·	·	·	·	·	3	·	·	2
**29**	·	·	·	·	·	·	·	·	·	·	1	·	·	·	·	·	·	·	·	13	·	·	·	·	·	·	·	·	23	·
**30**	·	·	·	·	·	·	·	·	·	·	·	·	·	·	·	·	·	·	·	·	·	·	·	·	·	·	·	5	·	·

Table S6: Rank order in which the top 15 most abundant clusters appear in each of the 20 independent *de novo* repeat assembly runs on data pooled from six species of Gomphocerine grasshoppers.

	Run																			
**Cluster**	**1**	**2**	**3**	**4**	**5**	**6**	**7**	**8**	**9**	**10**	**11**	**12**	**13**	**14**	**15**	**16**	**17**	**18**	**19**	**20**
**1**	1	1	1	1	1	1	1	1	1	1	1	1	1	1	1	1	1	1	1	1
**2**	3	3	3	2	3	2	3	2	2	3	2	2	2	2	3	2	2	2	2	2
**3**	2	2	2	3	2	3	2	3	3	2	3	3	3	3	2	3	3	3	3	3
**4**	4	4	4	4	4	4	4	4	4	4	4	4	4	4	4	4	4	4	4	4
**5**	5	5	5	5	5	5	5	5	5	5	5	6	5	6	5	5	5	5	5	5
**6**	6	6	6	6	6	6	6	6	6	6	6	7	6	5	6	6	6	6	6	6
**7**	7	7	7	7	7	8	7	7	7	7	7	8	7	7	7	7	7	7	8	7
**8**	8	8	8	8	8	9	8	8	8	8	8	9	8	8	8	8	8	8	9	8
**9**	9	10	9	9	10	10	9	9	9	10	9	11	11	11	9	9	9	9	10	9
**10**	10	11	11	10	11	11	10	10	10	12	11	12	13	12	11	12	10	11	11	11
**11**	19	12	13	11	9	19	12	11	13	9	10	10	12	10	10	11	19	10	12	19
**12**	12	14	12	13	12	13	13	12	11	14	12	13	14	13	12	13	11	12	13	12
**13**	11	13	14	12	13	12	11	13	12	15	13	14	15	14	13	14	12	13	14	13
**14**	14	15	15	14	14	14	15	14	14	16	15	16	16	15	14	15	13	15	7	14
**15**	13	16	16	16	15	16	14	15	20	18	14	17	19	17	16	16	16	17	16	15

Table S7: Annotation of repeat clusters using RepeatMasker with the Metazoan database of repeats. The table shows all cases with more than 1% hits.

Cluster	Class	Family	# Hits	Hits [%]
1	Satellite	Satellite	108,700	92.3%
2	DNA	DNA	13,135	20.7%
	RC	Helitron?	7,849	15.1%
	RC	Helitron	6,216	8.7%
	DNA	hAT-Charlie	2,281	3.2%
	Unknown	Unknown	1,291	1.6%
3	-	-	-	all <1.0%
4	RC	Helitron	28,700	72.0%
	Unknown	Unknown	704	1.5%
	DNA	hAT-Ac	888	2.0%
	Unknown	Unknown	704	1.5%
5	LINE	CR1	21,554	95.3%
6	RC	Helitron	14,331	54.7%
	DNA	hAT-Tip100	2,404	9.2%
	LINE	CR1	1,413	3.4%
	DNA	hAT-Blackjack	491	1.4%
7	RC	Helitron	5,130	27.7%
8	RC	Helitron	10,494	46.7%
	DNA	TcMar-Tc1	5,405	15.5%
9	LINE	I	15,349	96.7%
10	tRNA	tRNA	1,088	2.37%
11	RC	Helitorn	12,521	55.6%
12	LINE	CR1	13,919	92.8%
13	LINE	BovB	13,572	95.7%
14	LINE	BovB	10,994	75.7%
15	-	-	-	all <1.0%

Table S8: Pearson correlation of genome size with repeat abundance for the first 15 clusters. Repeat abundance and genome size has been estimated for nodes by ancestral state reconstruction (as for Figure S12).

Cluster	Annotation	*r*	*t*	df	*p*
1	Satellite	0.87	5.19	9	0.00057
2	Helitron	0.12	0.37	9	0.72
3	unknown	0.26	0.82	9	0.43
4	Helitron	0.23	0.71	9	0.49
5	LINE-CR1	-0.40	-1.31	9	0.22
6	Helitron	-0.38	-1.23	9	0.25
7	Helitron	0.88	5.66	9	0.00031
8	Helitron	-0.57	-2.11	9	0.064
9	LINE1	0.30	0.94	9	0.37
10	unknown	0.64	2.51	9	0.033
11	Helitron	0.04	0.13	9	0.90
12	LINE-CR1	0.34	1.08	9	0.31
13	LINE BoV-B	0.02	0.06	9	0.95
14	LINE BoV-B	0.33	1.04	9	0.32
15	Simple repeat	0.94	8.48	9	0.000014

Figure S1: Flow chart of the data processing procedure. Each species is symbolized by a different shade of grey and the two sexes are symbolized by filled and hatched texture. Horizontal lines symbolize subsets of the data.

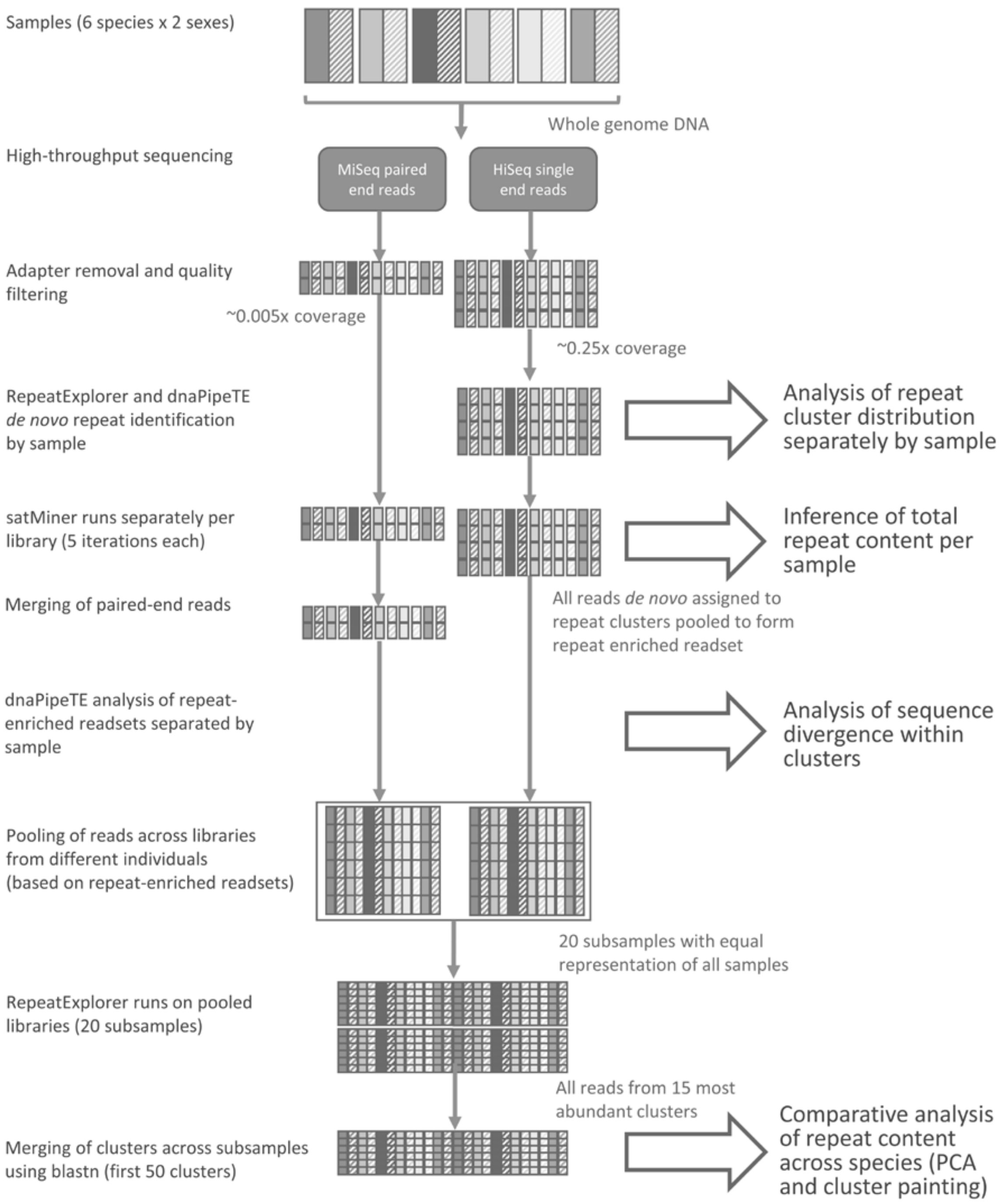

Figure S2: Genome size as determined by flow cytometry based on three males per species using a single *Acheta domesticus* male (diamonds) as a size standard (horizontal line shows the mean value across all *Acheta domesticus* measurements). Flow cytometry signals were converted to genome sizes by regression (Figure S23). rCV varied between 3.8 and 7.4 per sample with a mean of 3.9-7.0 per species (lowest in *Chorthippus biguttulus*, highest in *Gomphocerus sibiricus*). Numbers above data points show average values (± SE) per species.

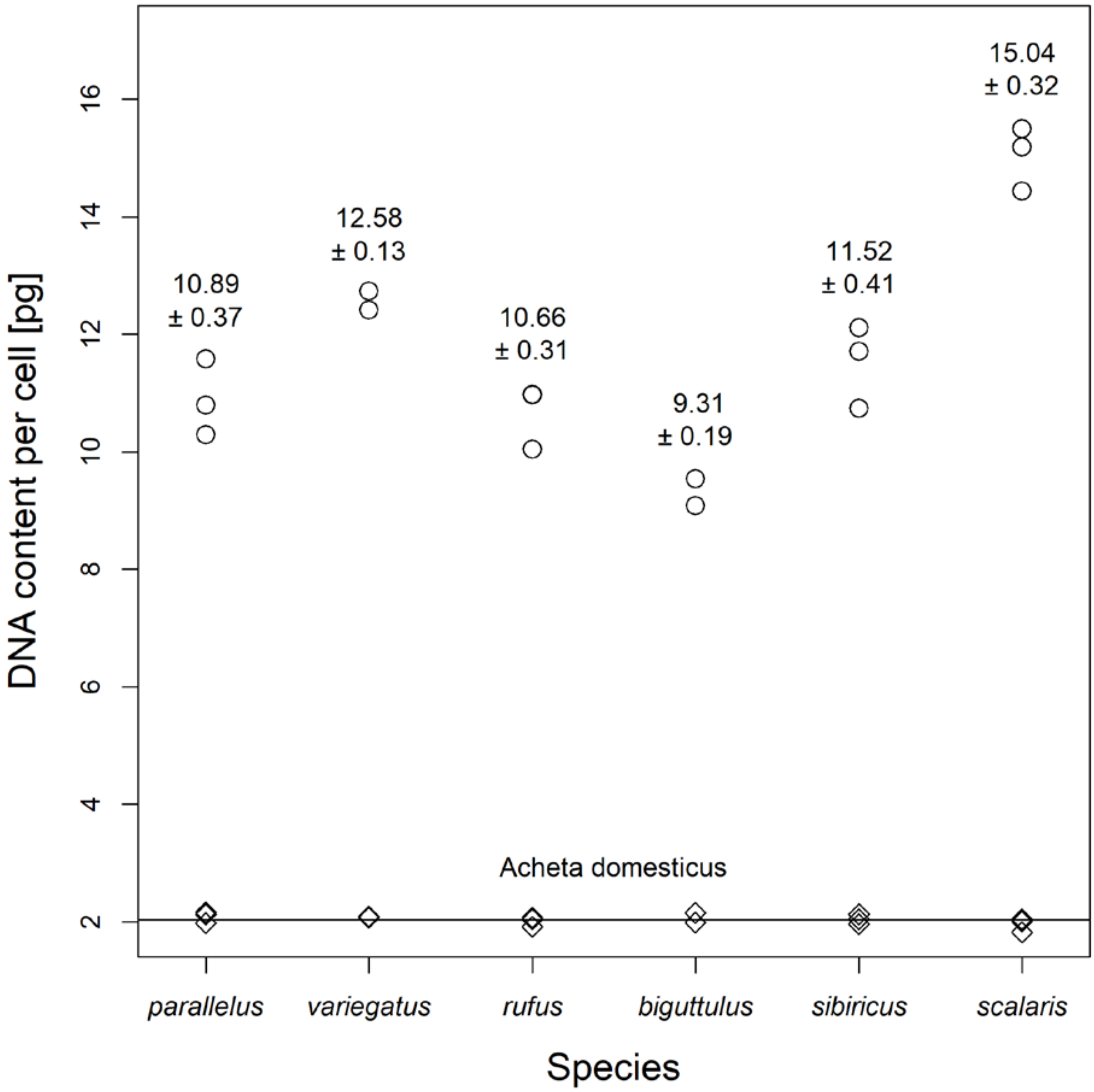

Figure S3: Repeat content estimation based on multiple satMiner iterations. Dashed lines show the proportion of reads (p_i in main text) *de novo* assigned to repeat clusters in each iteration *i*. Dotted lines show the proportion of reads identified as repeats (based on *de novo* clustering of a subset in combination with querying the main pool, q_i in main text). Solid lines show the sum $p_i + q_i$ of the proportion of reads already identified as repeats and the fraction identified as repeats in a subset of the remaining reads. Numbers in the upper left corner show the cumulative estimate of repeat content in the final iteration. Numbers in the lower right corner show the total number of contigs assembled by satMiner. Results for females are shown in black and results for males in grey.

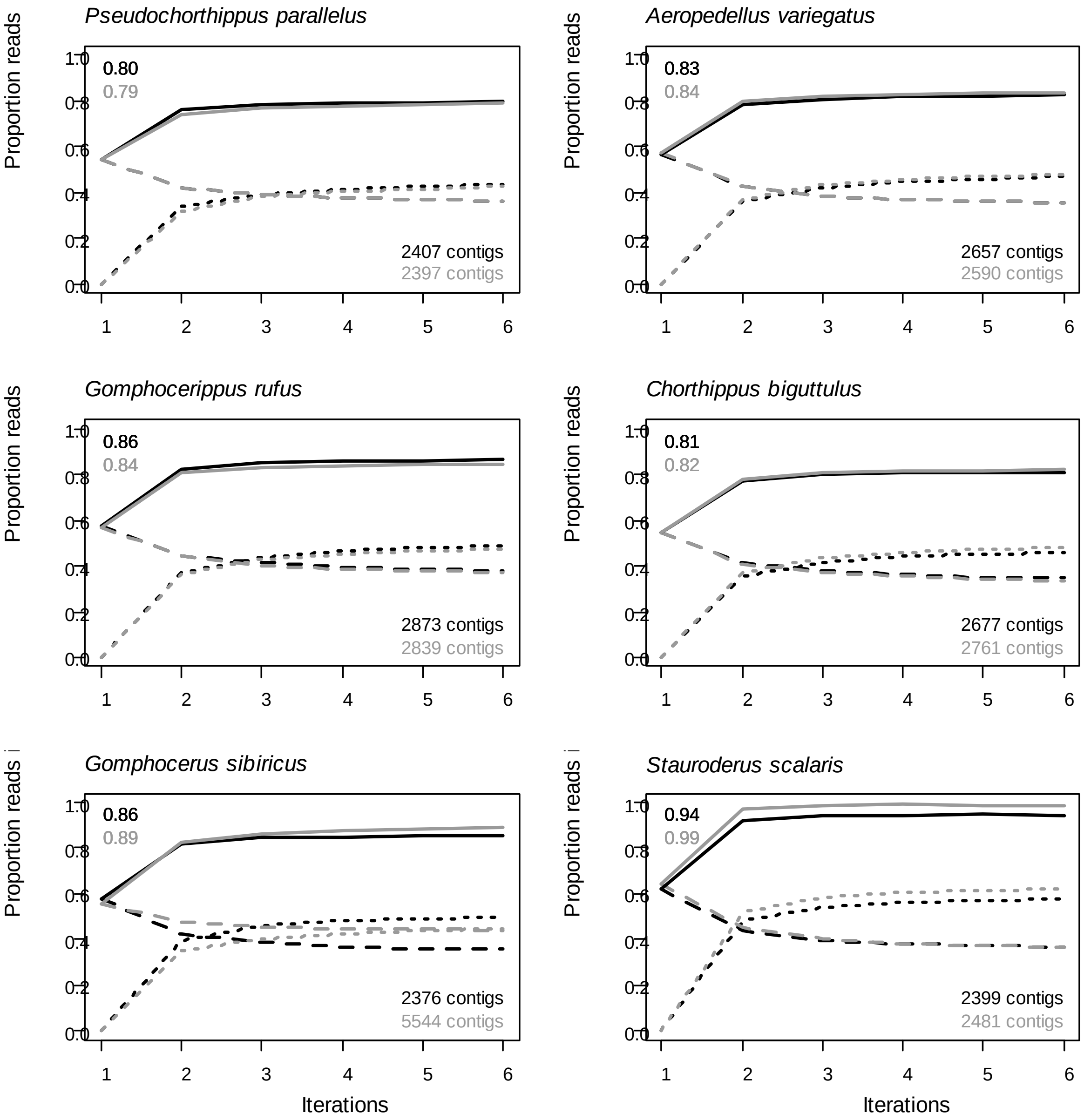

Figure S4: Distribution of repetitive DNA across repeat clusters annotated by RepeatMasker for six species of grasshopper. The data show results from a single female per species.

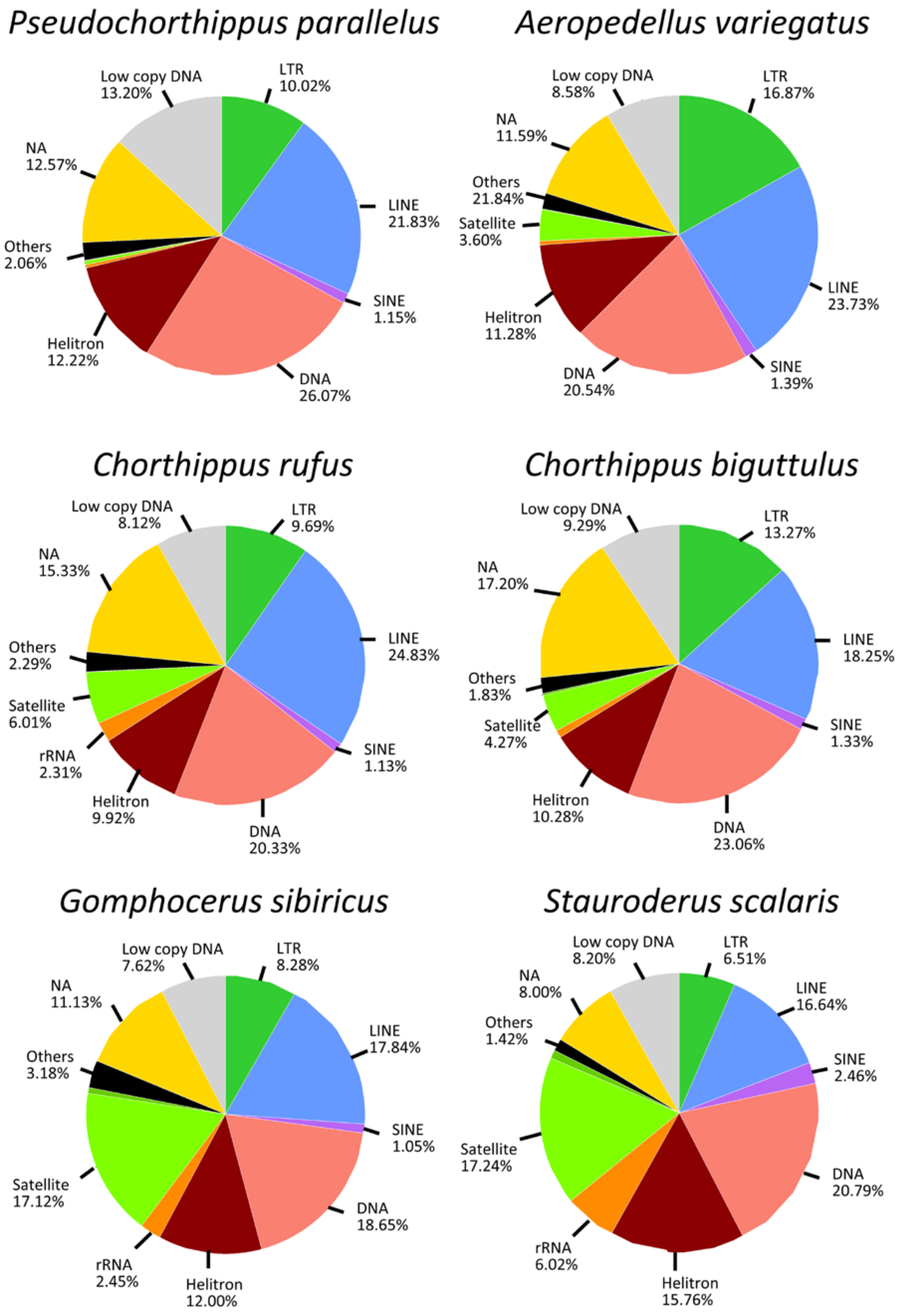

Figure S5: Distribution of repetitive DNA across repeat clusters annotated by RepeatMasker for six species of grasshopper. The data show results from a single male per species.

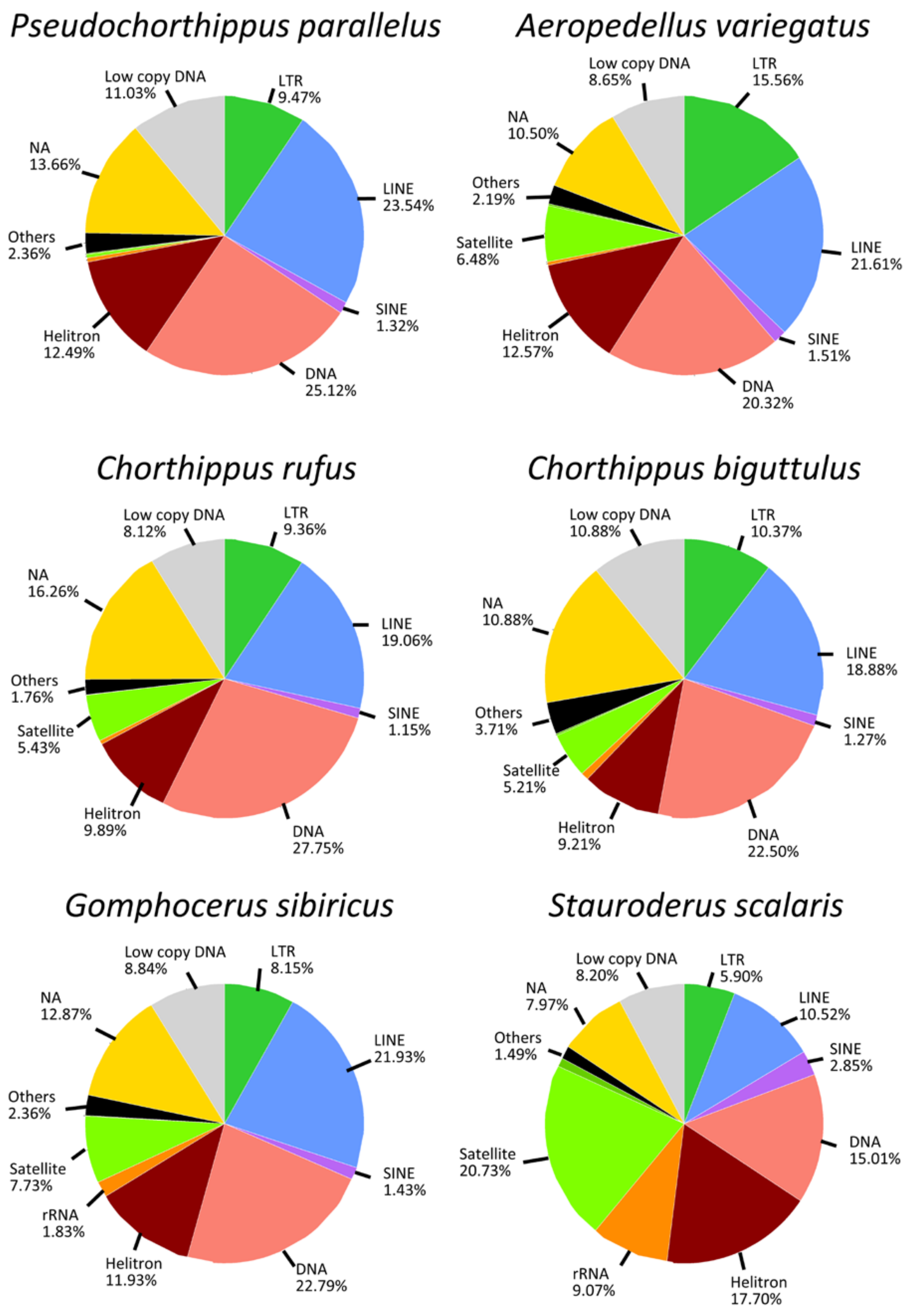

Figure S6: Distribution of *de novo* assembled repeat clusters for a *Locusta migratoria* female. Results are based on a single RepeatExplorer run. The vertical line shows the repeat content as estimated by RepeatExplorer based on this single run.

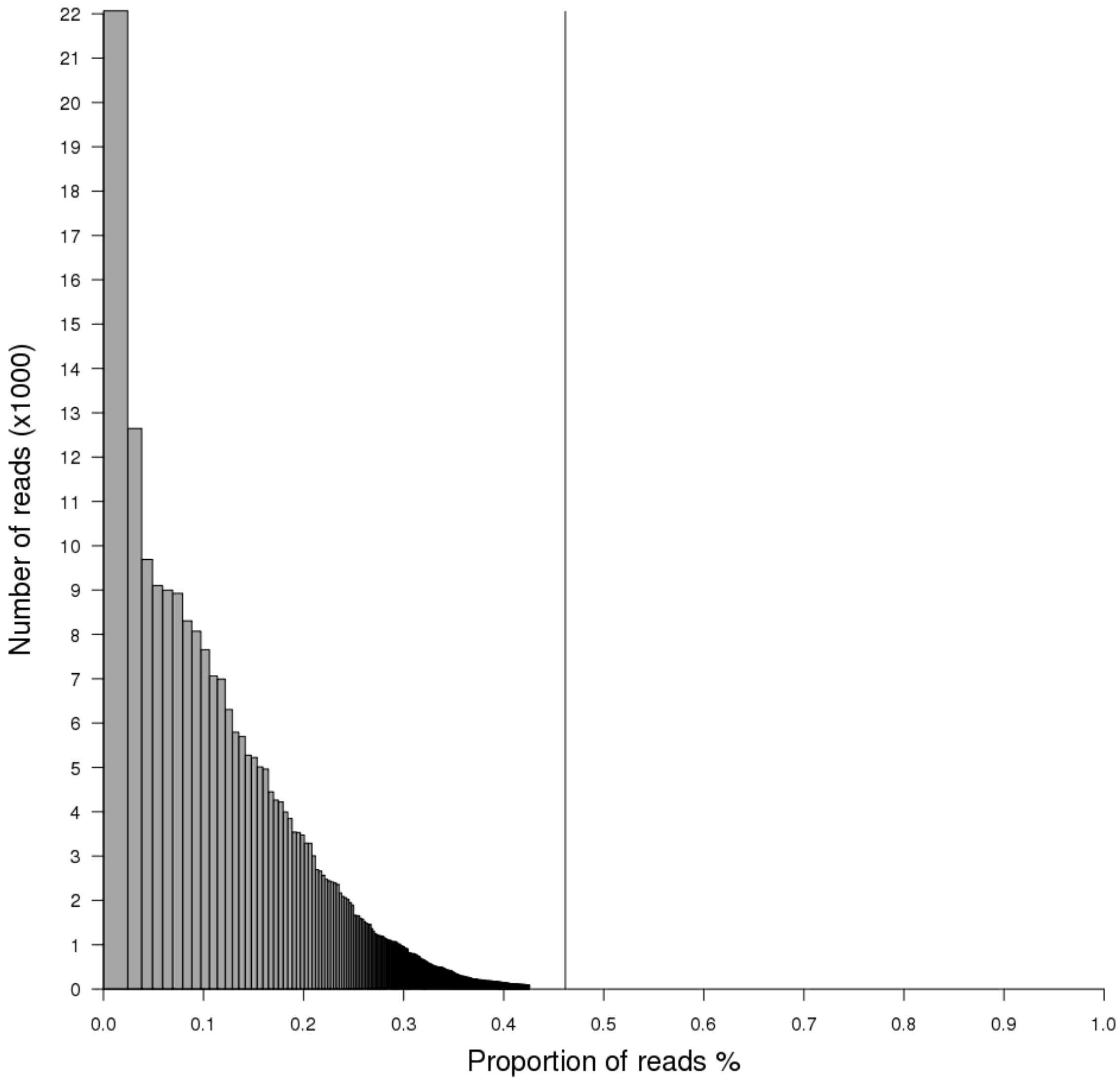

Figure S7: Divergence distribution within clusters of repetitive DNA in six species of grasshoppers across repeat-envirched datasets. The data show results from a single female per species.

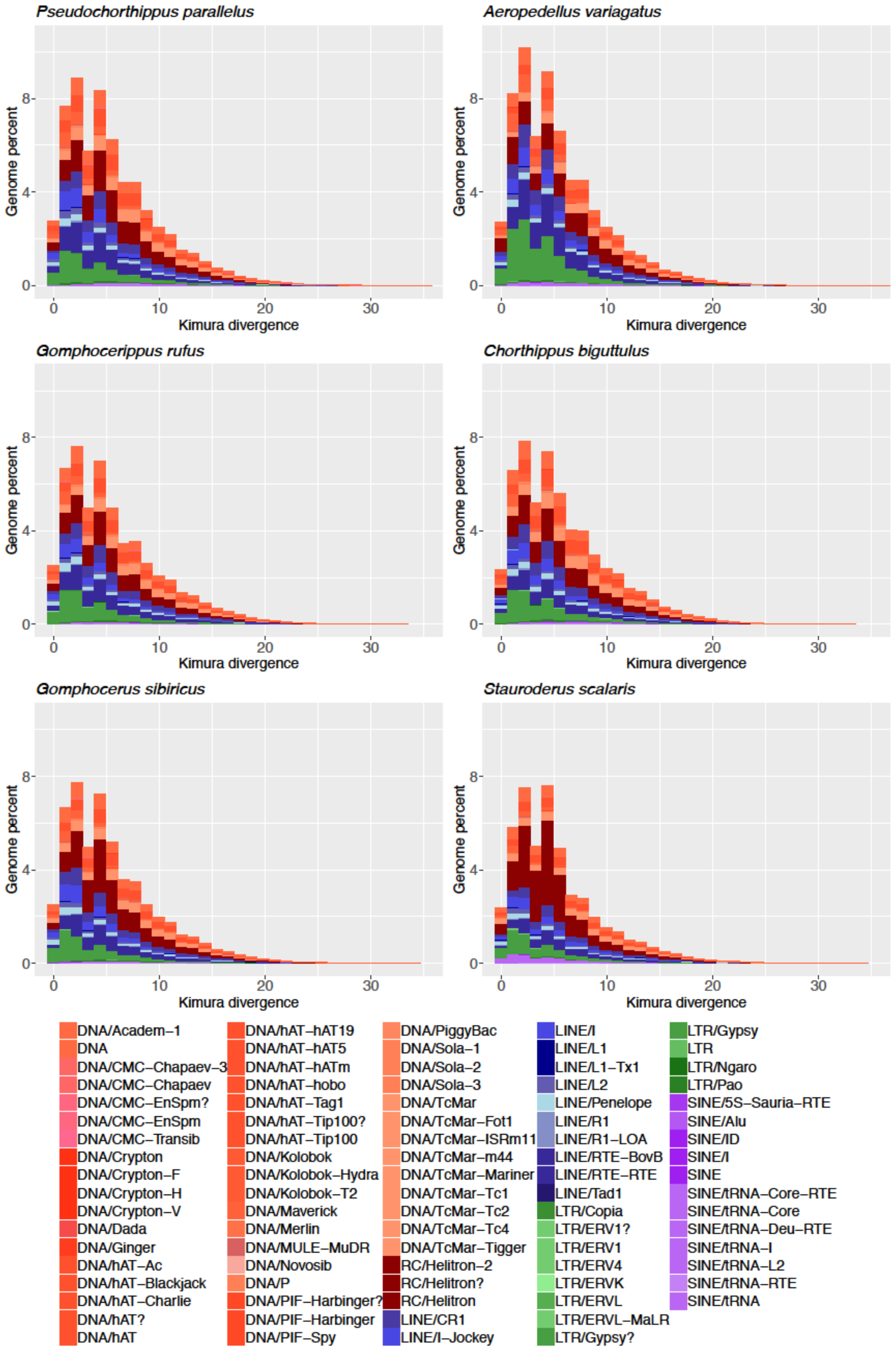

Figure S8: Divergence distribution within clusters of repetitive DNA in six species of grasshoppers across repeat-envirched datasets. The data show results from a single male per species.

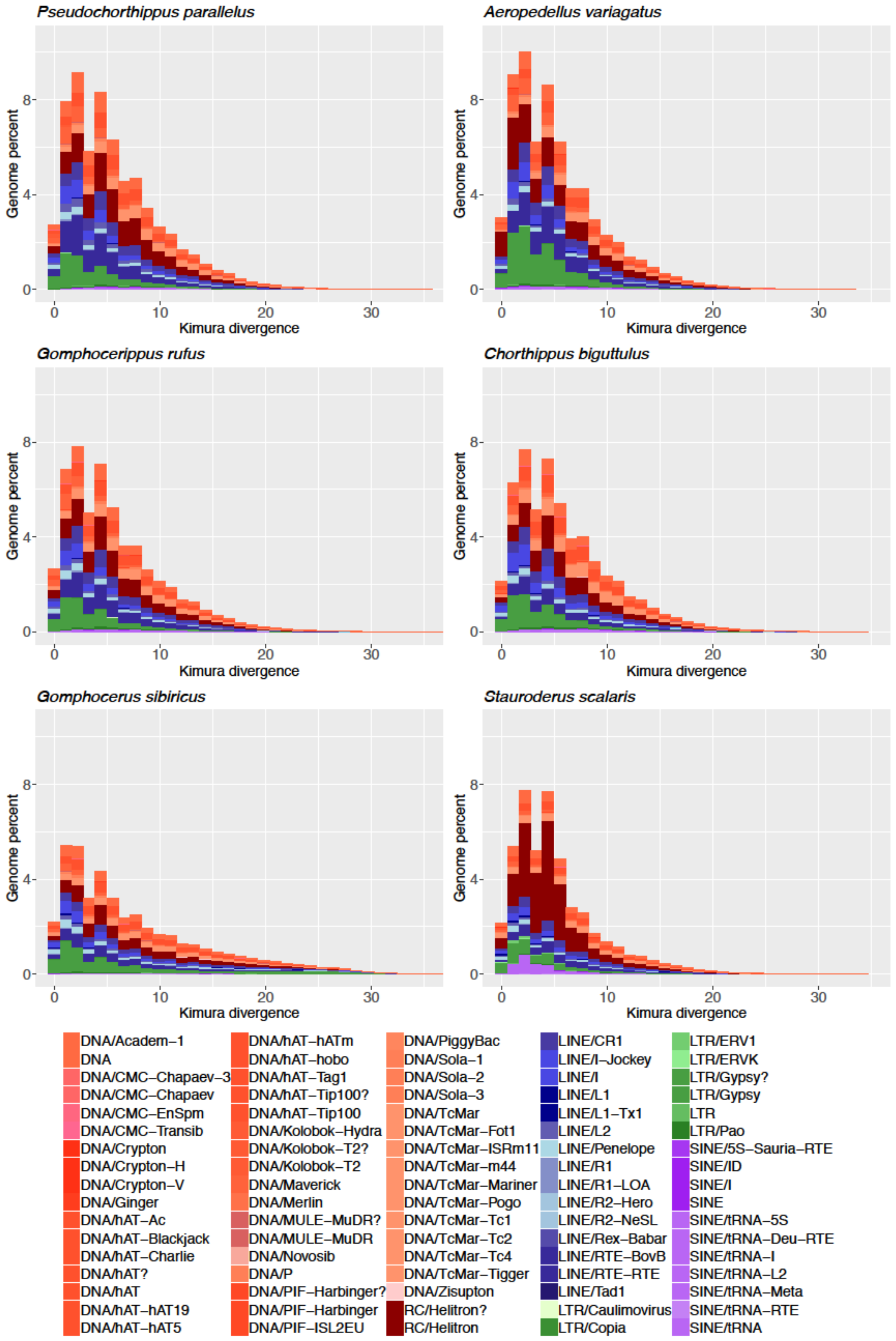

Figure S9: Correlation of repeat cluster size across the sexes. Results are shown across the first 10 repeat clusters with different clusters shown by different symbols. Each data point refers to a single cluster for a single species with six data points per cluster and thin lines showing the major axis regression line for each cluster. The dashed grey line shows the line of equal cluster size in females and males.

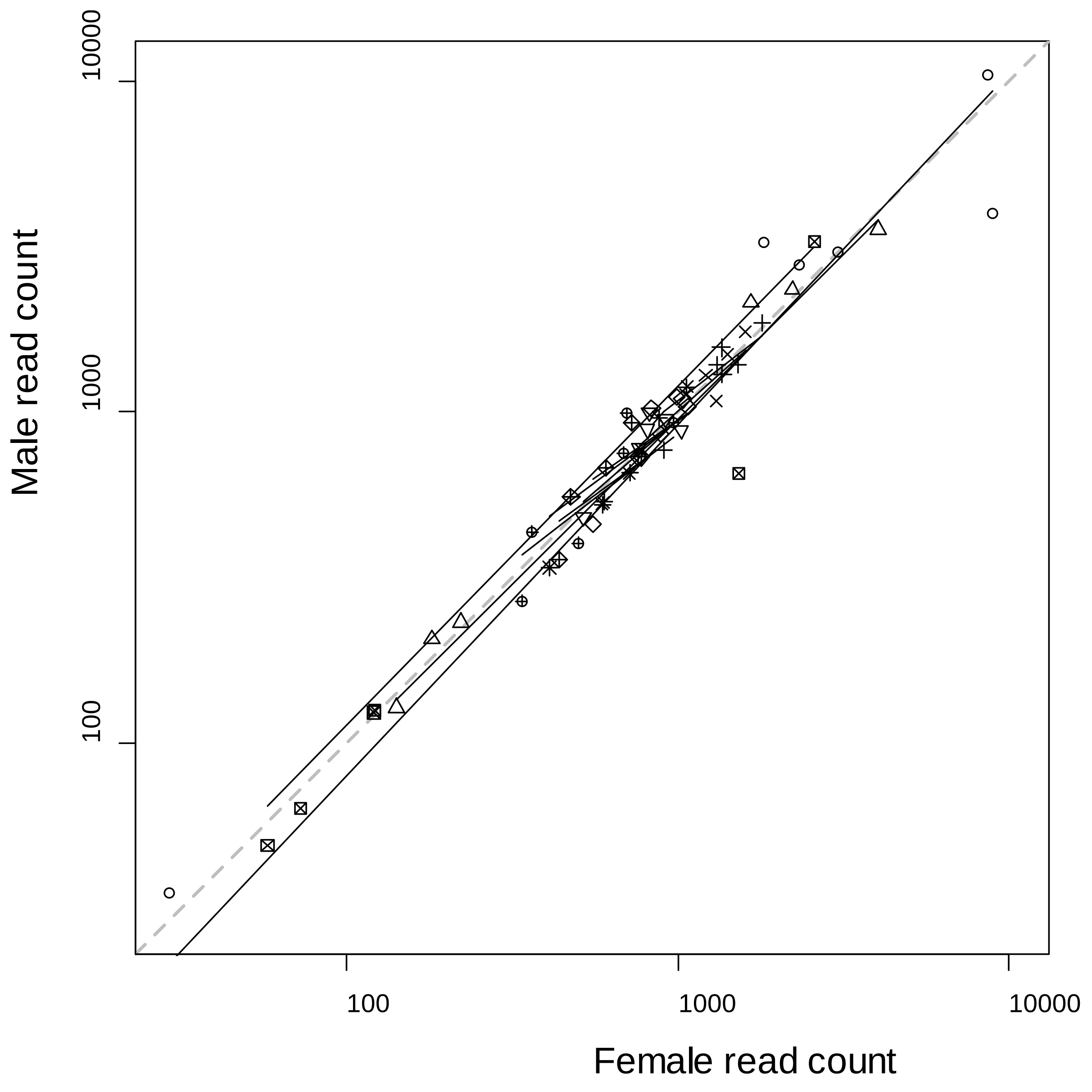

Figure S10: Sample specific phylogeny based on COI, COII and COIII mitrochondrial genes.

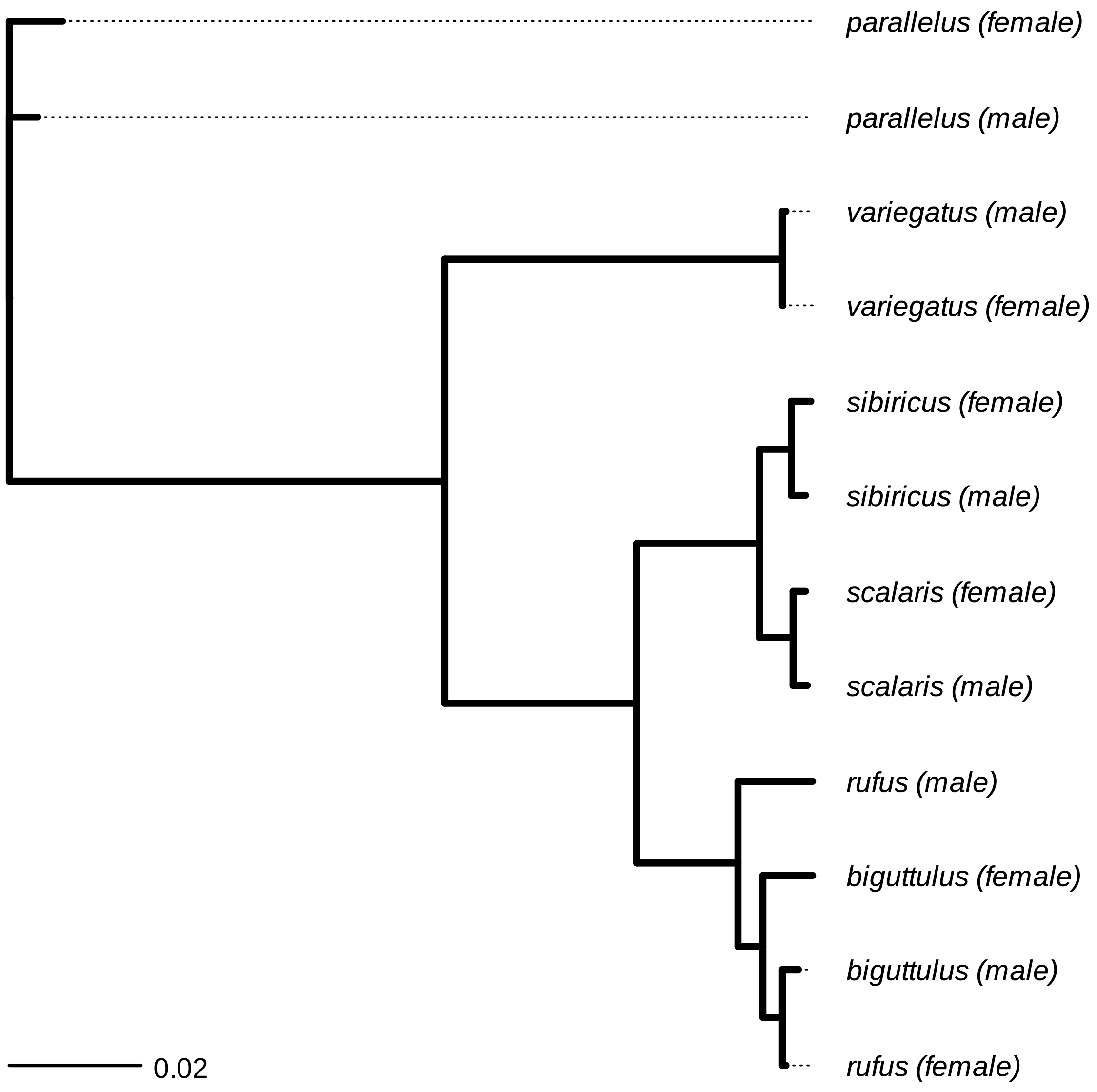

Figure S11: Sample-specific unrooted gene trees by mitochondrial gene locus.

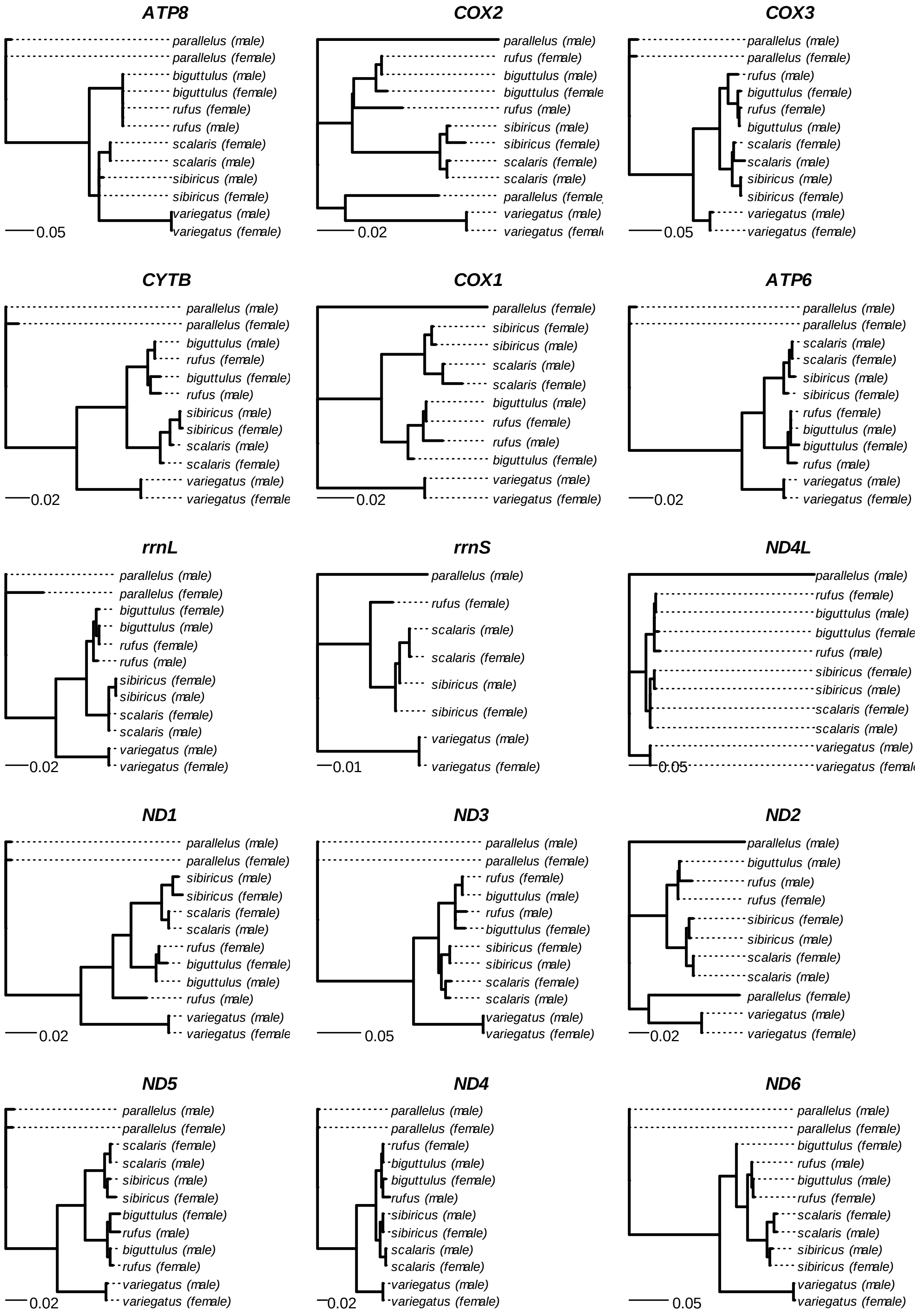

Figure S12: Repeat cluster size changes across the phylogeny. Grey dots show estimated abundance of reads (standardized to percentage of the largest node within a cluster). Edge width shows the proportional change (black = increases, red = decreases). Correlations of genome size with repeat abundances are shown in Table S8.

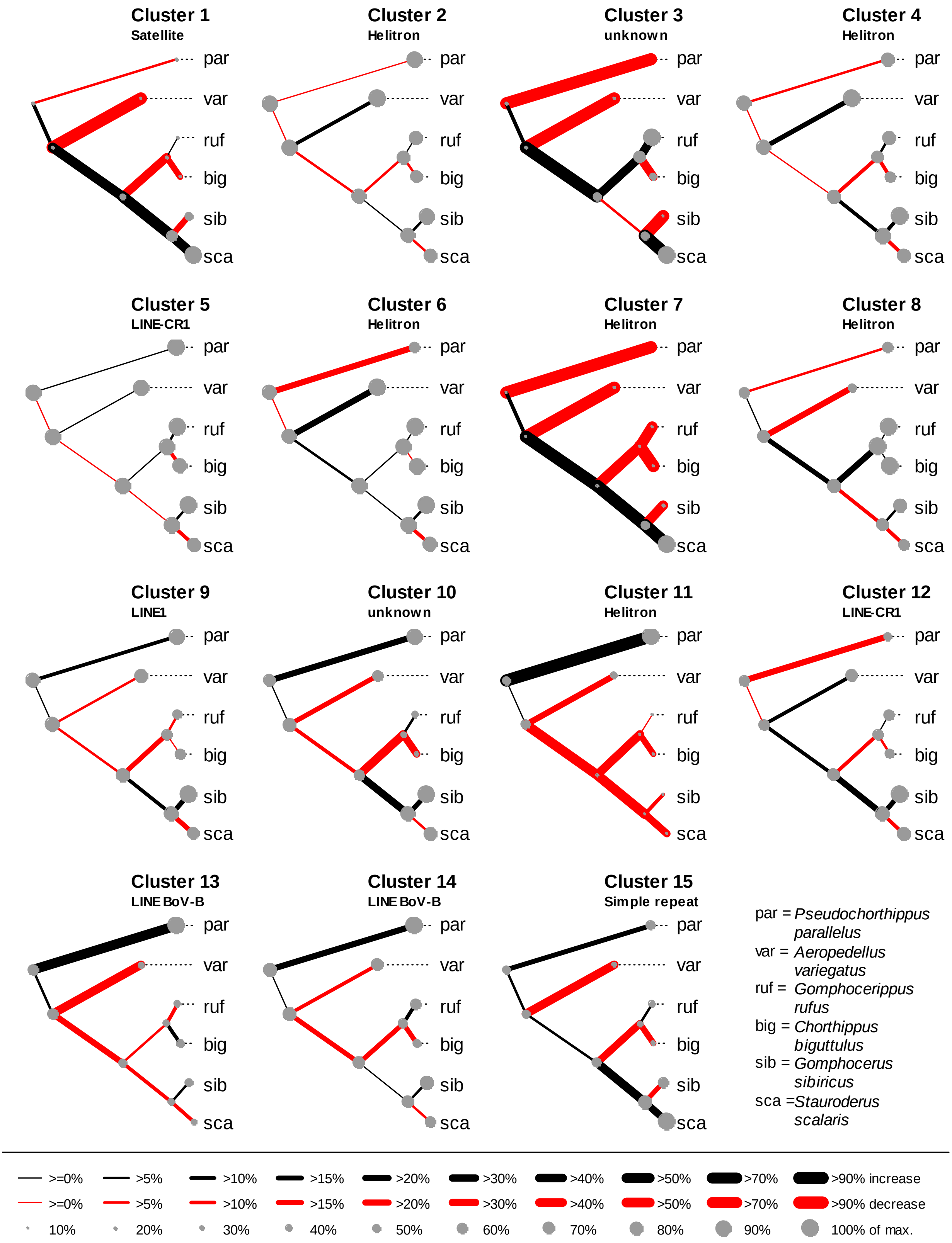

Figure S13: Cluster-paining approach to species-specific differences within cluster. The plot shows the four largest cluster with dots representing reads and read overlap by edges. The six different species are shown by different colours.

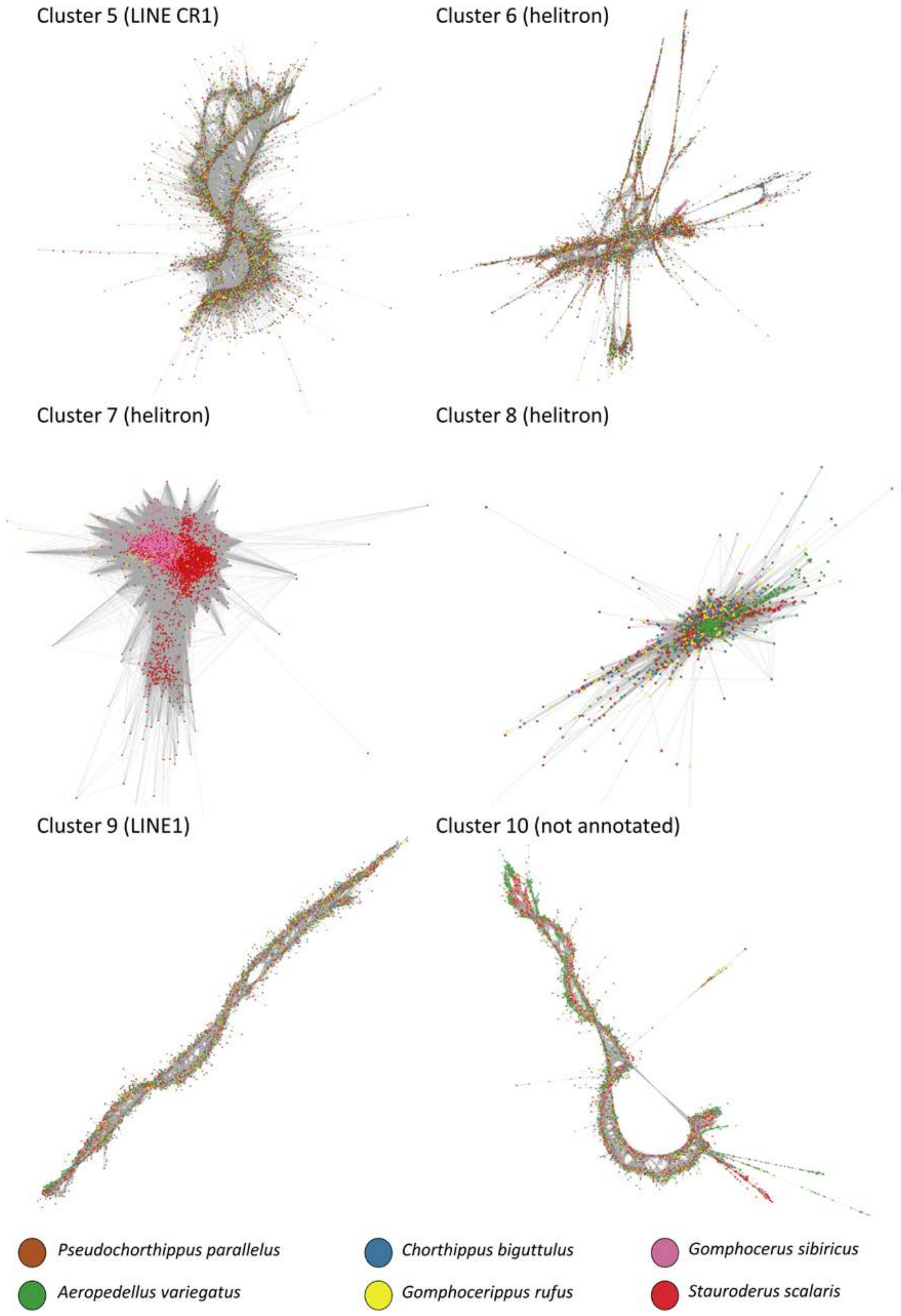

Figure S14: Calibration of flow cytometric signal intensity to genome sizes based on regression of published genome sizes (Table S3) and signal intensity. Published genome sizes are available for only four species (dom = *Acheta domesticus*, par = *Pseudochorthippus parallelus*, sib = *Gomphocerus sibiricus*, sca = *Stauroderus scalaris*, Table S3). Furthermore, we used the published genomes of *Chorthippus brunneus* as a substitute for the very closely related *Chorthippus biguttulus* (big/bru).

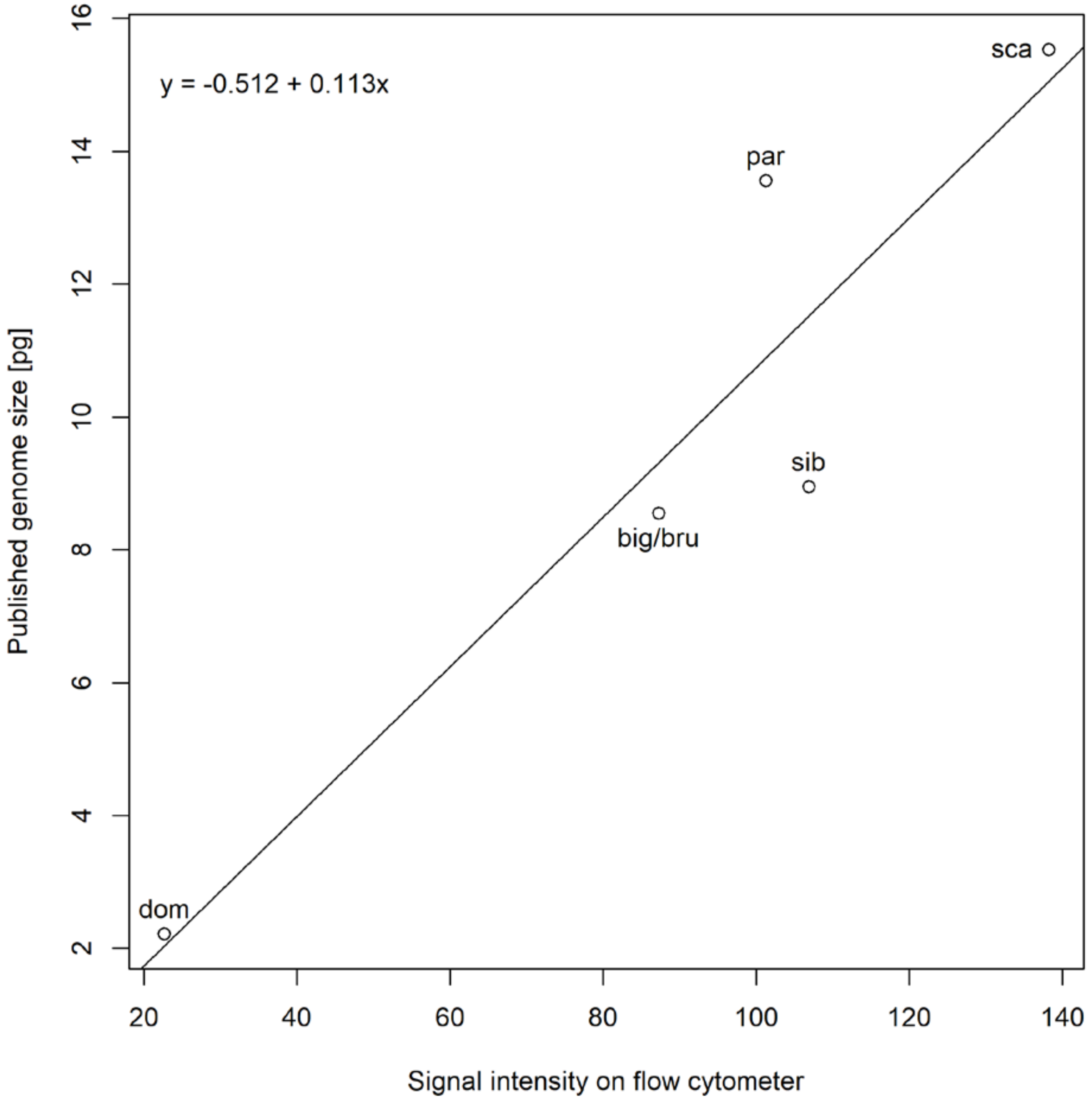

Supplementary references

Belda JE, Cabrero J, Camacho JPM, Rufas JS (1991) Role of C-heterochromatin in variation of nuclear DNA amount in the genus *Chorthippus* (Orthoptera, Acrididae). *Cytobios* **67**, 13-21.

Gosalvez J, Lopezfernandez C, Esponda P (1980) Variability of the DNA content in five Orthopteran species. *Caryologia* **33**, 275-281.

John B, Hewitt GM (1966) Karyotype stability and DNA variability in Acrididae. *Chromosoma* **20**, 155-172.

Petitpierre E (1996) Molecular cytogenetics and taxonomy of insects, with particular reference to the coleoptera. *International Journal of Insect Morphology & Embryology* **25**, 115-134.

Wang XH, Fang XD, Yang PC*, et al.* (2014) The locust genome provides insight into swarm formation and long-distance flight. *Nature Communications* **5**, 2957.

Wilmore PJ, Brown AK (1975) Molecular poperties of Orthopteran DNA. *Chromosoma* **51**, 337-345.
